# Hot-spot identification on a broad class of proteins and RNA suggest unifying principles of molecular recognition

**DOI:** 10.1371/journal.pone.0183327

**Published:** 2017-08-24

**Authors:** John L. Kulp, Ian S. Cloudsdale, John L. Kulp, Frank Guarnieri

**Affiliations:** 1 Conifer Point Pharmaceuticals, Doylestown, Pennsylvania, United States of America; 2 Department of Chemistry, Baruch S. Blumberg Institute, Doylestown, Pennsylvania, United States of America; 3 PAKA Pulmonary Pharmaceuticals, Acton, Massachusetts, United States of America; Universita degli Studi di Roma Tor Vergata, ITALY

## Abstract

Chemically diverse fragments tend to collectively bind at localized sites on proteins, which is a cornerstone of fragment-based techniques. A central question is how general are these strategies for predicting a wide variety of molecular interactions such as small molecule-protein, protein-protein and protein-nucleic acid for both experimental and computational methods. To address this issue, we recently proposed three governing principles, (1) accurate prediction of fragment-macromolecule binding free energy, (2) accurate prediction of water-macromolecule binding free energy, and (3) locating sites on a macromolecule that have high affinity for a diversity of fragments and low affinity for water. To test the generality of these concepts we used the computational technique of Simulated Annealing of Chemical Potential to design one small fragment to break the RecA-RecA protein-protein interaction and three fragments that inhibit peptide-deformylase via water-mediated multi-body interactions. Experiments confirm the predictions that 6-hydroxydopamine potently inhibits RecA and that PDF inhibition quantitatively tracks the water-mediated binding predictions. Additionally, the principles correctly predict the essential bound waters in HIV Protease, the surprisingly extensive binding site of elastase, the pinpoint location of electron transfer in dihydrofolate reductase, the HIV TAT-TAR protein-RNA interactions, and the MDM2-MDM4 differential binding to p53. The experimental confirmations of highly non-obvious predictions combined with the precise characterization of a broad range of known phenomena lend strong support to the generality of fragment-based methods for characterizing molecular recognition.

## Introduction

The nature of how small molecules bind to proteins continues to be the object of many experimental[1–6] and theoretical[7–9] studies. Early investigations into how diverse small organic compounds, which are commonly referred to as fragments, collectively bind to localized sites on proteins using X-ray crystallography[10, 11] or NMR[12] were originally developed as new methods for generating lead compounds in drug design. The overlapping and adjacent binding of fragments suggests how these entities might be chemically linked into larger higher affinity molecules—a necessary process for lead generation since fragments usually bind to proteins with very low affinity,[13, 14] often in the millimolar range. Fragment-based approaches have produced drug candidates, validating the clinical relevance[15, 16] of these methods. They have also made fundamental contributions to the basic understanding of protein-protein[17–23] interactions (PPI). PPIs often have long extensive interfaces that enable a multitude of potentially stabilizing interactions spread across a vast surface. Studies of fragment-protein interactions, however, indicate that in many cases the association free energy responsible for PPIs occurs at highly localized positions within the interface: so called “hot spots”. This finding not only furthers our basic understanding of PPIs, but pragmatically indicates that drugs designed to break PPIs should be targeted to these sites. Thus, fragment-based approaches have now become important methods in both pharmaceutical and academic research.

Experimental fragment-based methods are extremely resource intensive and thus it would be highly desirable to perform complimentary computer simulations that could assist with focusing and minimizing such experiments. These considerations led to the development of the GRID[24] and MCSS[25] algorithms for studying protein-fragment interactions. The realization, however, that these methods incorrectly overestimate the number of hot spots on proteins motivated Vajda[26] to develop the “solvent mapping” technique, which has proven to be highly accurate in identifying protein hot spots in a range of important[27–32] studies. Solvent mapping is a heuristic algorithm that enhances the sampling of fragments over the whole protein surface by initially zeroing out the van der Waals term and part of the solvation energy. This has the effect of dramatically smoothing out the protein surface, which eliminates a large number of small local minima that a probe fragment could inadvertently get stuck in. After thousands of potential fragment-protein interaction sites are located with a multi-start simplex method, a second minimization with van der Waals interactions and all solvation terms turned on is performed, which now recreates a realistic representation of the protein surface, to determine the multitude of final positions for a single fragment. The last step is to identify and energy rank localized sites on the macromolecule predicted to have high affinity for a broad diversity of fragments, the so-called clustering step.

A compelling alternative to an *ad hoc* method like solvent mapping would be to generate a Boltzmann weighted distribution of fragment binding sites over the whole macromolecule with grand canonical Monte Carlo (GCMC), because this simulation produces the theoretically correct set of fragment-macromolecule binding free energies, not simply minimized enthalpies. The grand canonical ensemble probability density function—exp[- (ΔE+μN)/RT]–is the Boltzmann distribution augmented with the chemical potential (μ) that accounts for changing the number of particles (N) at fixed temperature (T) with R being the universal gas constant. Using a standard Monte Carlo algorithm, a fragment is randomly inserted or deleted into the protein simulation cell, the ΔE is computed for this change, and this new trial configuration is accepted or rejected with the grand canonical probability density function. When this process is repeated for millions of steps the formally correct distribution of fragment binding states is produced in the (V, T, μ) ensemble. This notation means that volume, temperature, and chemical potential are initially set and kept constant throughout the simulation. At a given fixed chemical potential GCMC commonly produces dozens of sites on the macromolecule that have high affinity for the fragment. The term affinity here means binding free energy (not enthalpy), because chemical potential is a formally correct free energy. GCMC has nothing to say, however, about relative binding affinities—all sites populated with fragments are deemed equally probable and thus there is no way of distinguishing which one of these sites on the macromolecule has higher or lower affinity for the fragment. Quantitatively predicting the rank order difference in binding free energy of these different and distinct populated sites can be done by running a sequence of GCMC simulations at successively decreasing values of the chemical potential—a technique known as Simulated Annealing of Chemical Potential[33] (SACP). As the chemical potential is gradually lowered, individual fragment-occupied sites are successively depopulated. The weaker binding sites are depopulated with a small lowering of the chemical potential while the stronger, higher affinity, binding sites require significantly more lowering of the chemical potential to depopulate. SACP gives the chemical potential when every particular site is vacated and thus a quantitative and formally correct free energy metric of the relative binding of a fragment to different sites on the macromolecule is obtained. Furthermore, SACP has proved to be especially valuable in understanding the hydration free energy on a macromolecular surface. Hydration free energy is especially difficult to predict, because it is often a multibody water-water-macromolecule phenomena. SACP is a uniquely powerful way of discovering multibody water-macromolecule interactions, which was first illustrated when the method was used to predict the differential hydration propensities between the major and minor grooves of DNA[33] in 1996.

**Fig 1:**
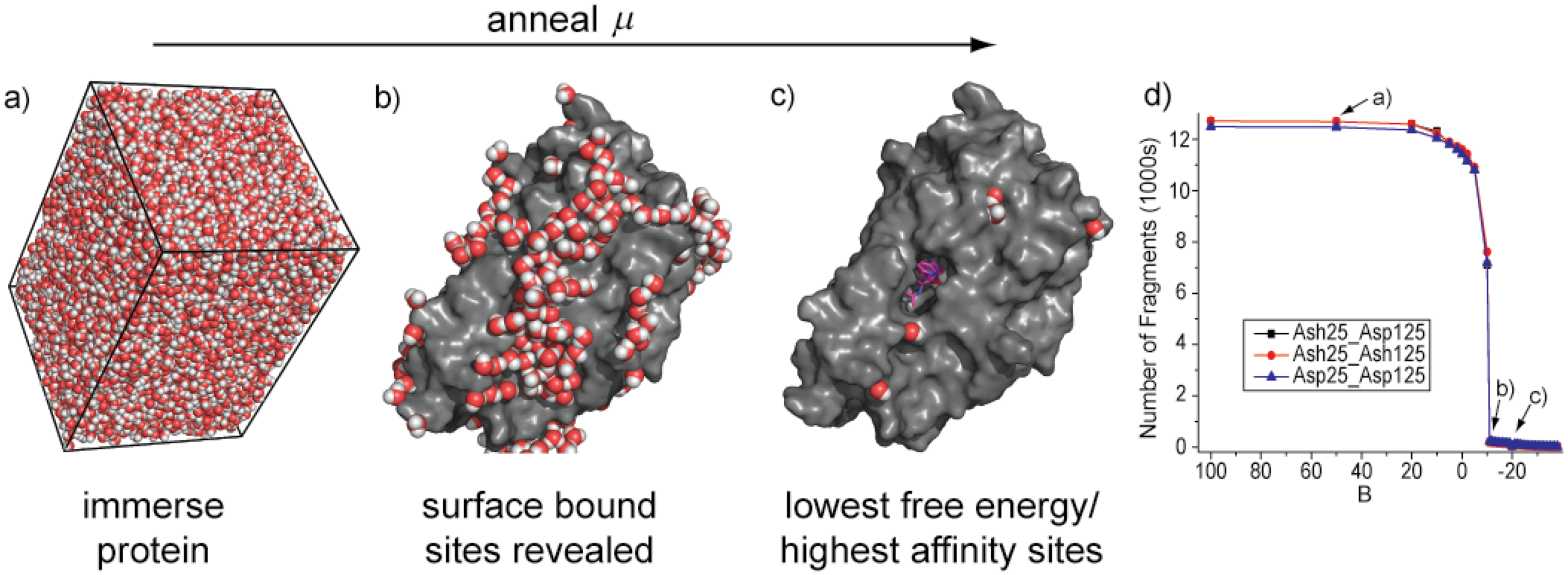
The chemical potential simulation. The protein is placed in a simulation box big enough to accommodate 3 layers of solvent—water in this case. The simulation is started with a high chemical potential and solvent molecules rapidly fill the box and occupy (a) all possible cavities at the surface and deep within the protein. As the chemical potential is lowered, not much happens until this free energy reaches a critical level that is capable of destroying the solvent cohesion and the system goes through a phase transition that evacuates the bulk solvent and most of the protein-bound solvent, exposing high affinity (b) binding sites. Continuing to lower the chemical potential further reveals the extremely high affinity (c) binding sites. A plot of the number of fragments in the simulation box versus the chemical potential shows the abrupt transition when the bulk solvent is evaporated from the cell. The charged state of the catalytic aspartates in HIV protease (Asp = charged, Ash = neutral) does not change the phase transition.

The results of a SACP simulation using water as the probe fragment are illustrated in Fig 1. At high values of the chemical potential insertion of water into the protein cavities and bulk has a high probability and the system becomes densely hydrated (Fig 1a). The chemical potential is lowered by a small amount until equilibrium is re-established. This process is repeated, resulting in very little change in the system, until a chemical potential that is low enough to break the water-water solvent cohesion occurs. At this point, the chemical potential probabilistically favors water deletion over water insertion and the system goes through a phase transition (Fig 1b) resulting in evacuation of almost all waters from the protein simulation cell (Fig 1c). As the chemical potential is lowered further even the identified tightly bound water molecules can be removed from the protein—the additional increment of chemical potential required to finally eliminate these waters indicates their relative free energy of binding.

There are several key benefits of this phase transition method: (i) the high positive starting value for the chemical potential causes the simulation cell to become densely packed, forcing fragments into all interstices of the protein, which efficiently samples multi-fragment configurations—particularly critical for water modeling; (2) as the chemical potential is annealed to lower values, the system goes through a phase transition where the regions of the simulation cell away from the protein surface are voided—the chemical potential at which this happens distinguishes strongly bound fragments from transient weakly interacting fragments; and (3) as the chemical potential is lowered beyond the phase transition, fragment binding sites become isolated. In this latter regime, fragments have a negligible interaction with other fragments, and the chemical potential—the average free energy per molecule—can then be used to characterize the free energy of the fragment binding in each individual site.

While SACP is a powerful foundation for accurately predicting relative fragment-macromolecule binding free energies, it requires additional principles for investigating general molecular recognition phenomena. We previously proposed three governing principles—accurate prediction of 1) small organic fragment macromolecule binding free energy, 2) hydration free energy, and 3) macromolecule sites that have high affinity for a diversity of organic fragments and low affinity for water.[34, 35] These would apply without regard to any particular method used: either experimental or computational. If these three principles can be accomplished with any fragment-based technique, then a wide variety of seemingly disparate molecule recognition phenomena could be characterized and predicted. SACP is one particular method for assessing these principles, because it is easily generalized[36] beyond investigating water interactions—the binding free energy of any organic fragment to any macromolecule can be determined with these simulations. Specifically, a set of fragments representing a large variety of diverse organic functional groups and water are simulated with SACP on a macromolecule, a cluster analysis is performed to find the localized sites of the macromolecule that have a high affinity for a diversity of fragments, and a cluster site is eliminated if water is also predicted to bind there with high affinity (so-called water exclusion principle). The clustering set of fragments contains 13 entities (S2 Fig). The set contains all small relatively flat fragments. There is a mixture of hydrophobic and hydrophilic fragments as well as a variety of hydrogen bond done and accept types. These properties of the clustering set map out the chemical properties of the various biomolecule surface. SACP has been successfully applied (i) to show how mitochondrial aspartate amino transferase moonlights[37] as a lipid transporter, (ii) to the binding site predictions[35] of diverse wild-type and mutated hen egg white lysozymes, (iii) how pregnenolone binds to and modulates the cannabinoid[38] receptor, (iv) to the design of a very small molecule erythropoietin[39] agonist, (v) p38[40, 41] non-ATP site antagonists, and (vi) a cancer drug discovery[42] program.

To demonstrate the generality of this method, we ran the SACP system on a diverse set of examples: elastase, HIV protease, dihydrofolate reductase, HIV TAT-TAR, MDM2-MDM4-p53, RecA, and peptide deformylase (PDF). The first[43] co-crystal of elastase was reported in 1976 with subsequent structures[44–46] all producing a similar ligand binding position. In 1994, new co-crystals[47] with ligands almost identical to previous studies revealed that the binding site was dramatically larger. By contrast dihydrofolate reductase has an obviously very large[48] binding site, which holds the ligand and co-factor, but functional analysis reveals that the electron transfer process occurs at a pinpoint location within the binding site. In HIV Protease, there is a key bound water[49] molecule in the center of the binding site and thus this is an important protein for testing SACP. The binding of MDM2 and MDM4 to p53 provides a stringent test of a method’s ability to distinguish between subtly different homologous protein-protein[50–55] interactions. Because p53 dysfunction occurs in more than[56] 50% of all cancers, the ability to dissect such interactions is vital for understanding carcinogenic transformation. TAR is an RNA domain[57, 58] present in all HIV transcripts. When the HIV TAT protein binds to TAR, transcription is dramatically enhanced, thus this protein-RNA interaction[59] is an appealing target for antiviral drugs. The RecA protein[60] is responsible for repairing damaged[61, 62] bacterial DNA. Dozens of RecA proteins interlock[63] and bind to mutated DNA as a homo-oligomer that performs ATP driven repair operations in a directed[61] low fidelity manner that gives rise to resistant mutations. RecA is not only responsible for drug-resistant mutagenesis, but also for the sharing of the mutated genes resulting in the spreading of the developed[64] resistance. Peptide deformylase is a target for the development of antibaterial[65] and anticancer agents[66, 67]. These systems were chosen for this study, because collectively they represent a vast diversity of molecular recognition phenomena and they are all historically well characterized.

## Experimental

### Protein structures

Protein structures were downloaded from the Protein Data Bank (PDB).[68] For HIV protease, the 1SDT structure[69] was run under three different protonation states: (i) both catalytic aspartate residues deprotonated, the apo state[70] (Asp25_Asp125), (ii) one aspartate residue protonated and the other deprotonated, the ligand bound state,[70] where Asp25 has a proton on OD2[71] (Ash25_Asp125), and (iii) both aspartate residues protonated (Ash25_Ash125). The antiviral drug, indinavir, was removed prior to simulation. For dihydrofolate reductase, three PDB structures were simulated: 1J3I, 1J3J, and 1J3K, which represent the wild type, double mutant and quad mutant structures, respectively, in *Plasmodium falciparum*.[72] The ligand was removed for each structure and NADPH was retained. In an additional simulation on 1J3I, both the ligand and NADPH were removed. For the elastase enzyme, the 1ELA structure[47] was selected the peptide ligand removed. For the p53-MDM2 enzyme, the 1T4F structure[73] was simulated with the peptide ligand removed. For the p53-MDMX enzyme, the 3FDO structure[74] was used with the peptide ligand removed. For the RNA structure, the 2KX5 structure[59]—HIV TAR RNA with the peptide mimetic of Tat protein removed—was simulated. For the RecA protein, the 1xms structure[75] was simulated using a single monomeric unit with the ATP analog removed. Peptide deformylase, PDF, was modeled from the 1G27 pdb structure. All of the structures were checked, and corrected, for missing atoms or residues with the Profix program.[76] Hydrogen atoms were added with the Reduce program.[77] Protein protonation states were determined with PROPKA[78–81] and applied unless otherwise noted.

### Simulated Annealing of Chemical Potential calculations (SACP)

Each protein was placed in a simulation box with minimal dimensions to accommodate at least three layers of fragments. Next, a grand canonical Monte Carlo method was used to calculate fragment distributions.[82] A chemical potential is imposed between the simulation box and an ideal gas reservoir. The fragments are repeatedly inserted and deleted until the system equilibrates at this chemical potential as indicated by the average number of fragments becoming stable.[15, 83] During the simulation, the chemical potential is annealed in a succession of steps.[33] Annealing is carried out by gradually lowering the parameter B = μ_ex_/kT + ln <N> where μ_ex_ is the excess chemical potential related to protein interactions and <N> is the average amount of fragment molecules in the simulation. The series of steps starts at B equal to +100.0 and there are no fragments in the simulation cell. A defining characteristic of SACP is the clear occurrence of a phase transition. At high chemical potential the entire simulation cell is completely packed with fragments bound in every crevice of the protein and forming three tight packed solvation layers. As the chemical potential is gradually lowered very little change occurs in the system and suddenly within a very small ΔB, the simulation cell becomes evacuated leaving only tightly bound protein fragments. Further lowering of the chemical potential reveals the relative free energy of binding between these bound fragments as they successively depopulate the protein. We use energy cutoffs instead of the traditional distance cutoffs by pre-computing an optimal cutoff distance for each fragment type based on the maximum interaction energy between any pair of fragment types or residues. 0.1 kT was the cutoff energy contribution applied. Various cutoff distances are applied for atom-atom models and less costly multipole models for residues or fragments. This method has been described in further detail elsewhere.[35, 84]

A simplified but representative SACP protocol is to generate a random number (RanX) between 0 and 1 and to insert a fragment into the protein simulation cell if RanX<0.5 or delete a fragment if RanX>0.5. In either case this is a trial move with attempted insertion assigned a probability of P = exp[-[E(n+1)-E(n)]/(RT)]*exp[B]/(N+1) and attempted fragment deletion a probability of P = N*exp[-[E(n-1)-E(n)]/(RT)]*exp[-B] where E(n) is the energy of the system before an insert of delete, E(n+1) is the energy of the system after a fragment is inserted, and E(n-1) is the energy of the system after a fragment has been deleted., R is the gas constant and T is the temperature in Kelvin. B is the chemical potential of the simulation cell containing the protein and the only adjustable parameter. Operationally, a random number (RanX) is generated between 0 and 1 and the inserted or deleted fragmented is accepted as the new configuration if P< = RanX. When B is set high, the probability of inserting a fragment into the protein simulation cell is dramatically enhanced resulting in saturation with fragments. As the chemical potential is gradually lowered by decreasing the value of B, fragments persist in the simulation cell until a low enough B-value occurs that causes a dramatic evacuation of the simulation cell with almost all fragments exiting. Generally about 5 million simulation steps are done at each B-value and the simulation is run for about 30 different B-values. Note, when using somewhat more sophisticated procedures such as Mezei’s cavity-bias[85] to determine where to insert fragments more efficiently, the probability equation has to be adjusted to maintain detailed balance, but this is a technical matter that does not change the conceptual process. Details of the compute time and hardware configurations can be found in the Supporting Information.

### Fragment clustering and water exclusion

Hot-spot prediction was done using a simple clustering algorithm. The algorithm for fragment clustering and water exclusion for hot-spot identification contains four parameters: (i) the number of different fragment types used, typically 10–15 probe fragments including some amino acid side chains (Nature’s fragments) and a few other organic molecules such as pyridine and furan (ii) a chemical potential energy past the phase transition for each fragment type; (iii) the chemical potential energy of waters, which is used to exclude fragments, and typically occurs at the phase transition; and (iv) a radius for water exclusion, so that any fragment within 1 Å of the oxygen atom of a bound water will be excluded. After applying the simple clustering algorithm, there will often be multiple clusters or hot spots. Post-processing the data, two additional parameters are varied: (i) chemical diversity, the number of fragment types in a fragment cluster, and (ii) cluster radius, a radius for fragment cluster assignment—the center of mass of one fragment to the center of mass to another fragment. Chemical diversity and cluster radius allow the identification of tightly clustered hot spots or hot spots that have a larger area, such as binding sites with multiple subpockets.

### Compounds and assays

6-hydroxydopamine was purchased from Sigma-Aldrich. RecA inhibition assays were run as previously described.^79^ Indole derivatives were synthesized as previously described^87^ and NMR plus MS characterization can be found in the supporting information. Peptide deformylase assays described in supporting information.

## Results

### The small fragment 6-hydroxydopamine potently inhibits the RecA-RecA protein-protein interaction

**Fig 2:**
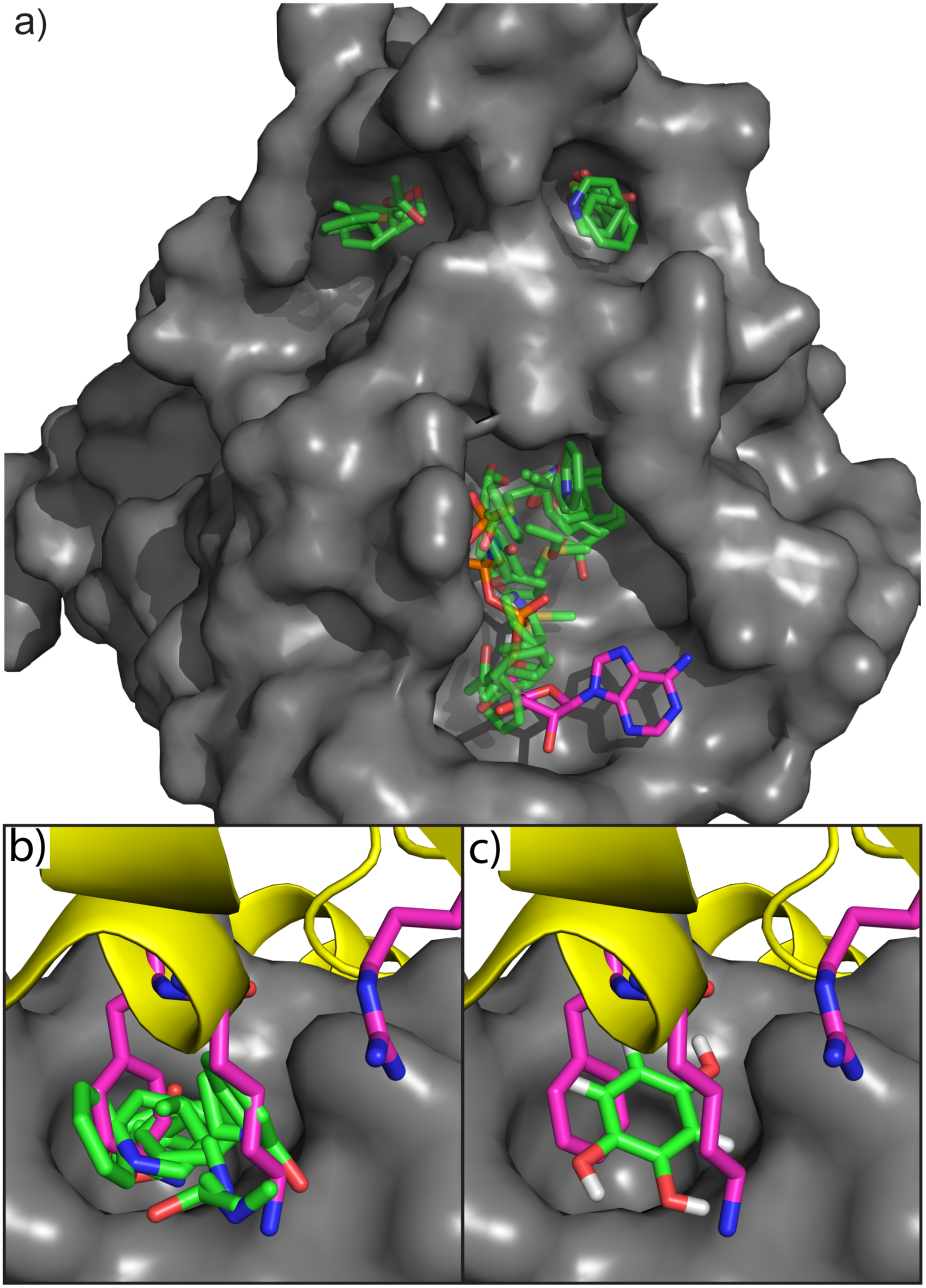
Hot spots on RecA. a) Sites where a diversity of fragments simultaneously binds with high affinity and where no high affinity waters appear. The sites correspond to the ATP binding site (ATP is shown in magenta), an interaction site between RecA monomers (top right site), and the DNA binding site (upper left site). Three universally conserved residues (K216, F217, and R222) from the neighboring subunit (yellow ribbons) are shown with magenta carbons and the interactions at the RecA-RecA site for these residues are shown in (b). Mutations of these three residues will result in the loss of RecA function, although F217Y results in a 250-fold increase in the interaction between RecA subunits. SACP predicts that alkylamines bind in the RecA pocket mimicking K216 interactions (b) and that phenol mimics the F217 interaction. SACP also predicts that adding extra hydroxyl groups to the benzene ring results in a higher affinity (c). When the fragment patterns are combined, SACP creates 6-Hydroxydopamine in the RecA-RecA interaction site.

**Fig 3:**
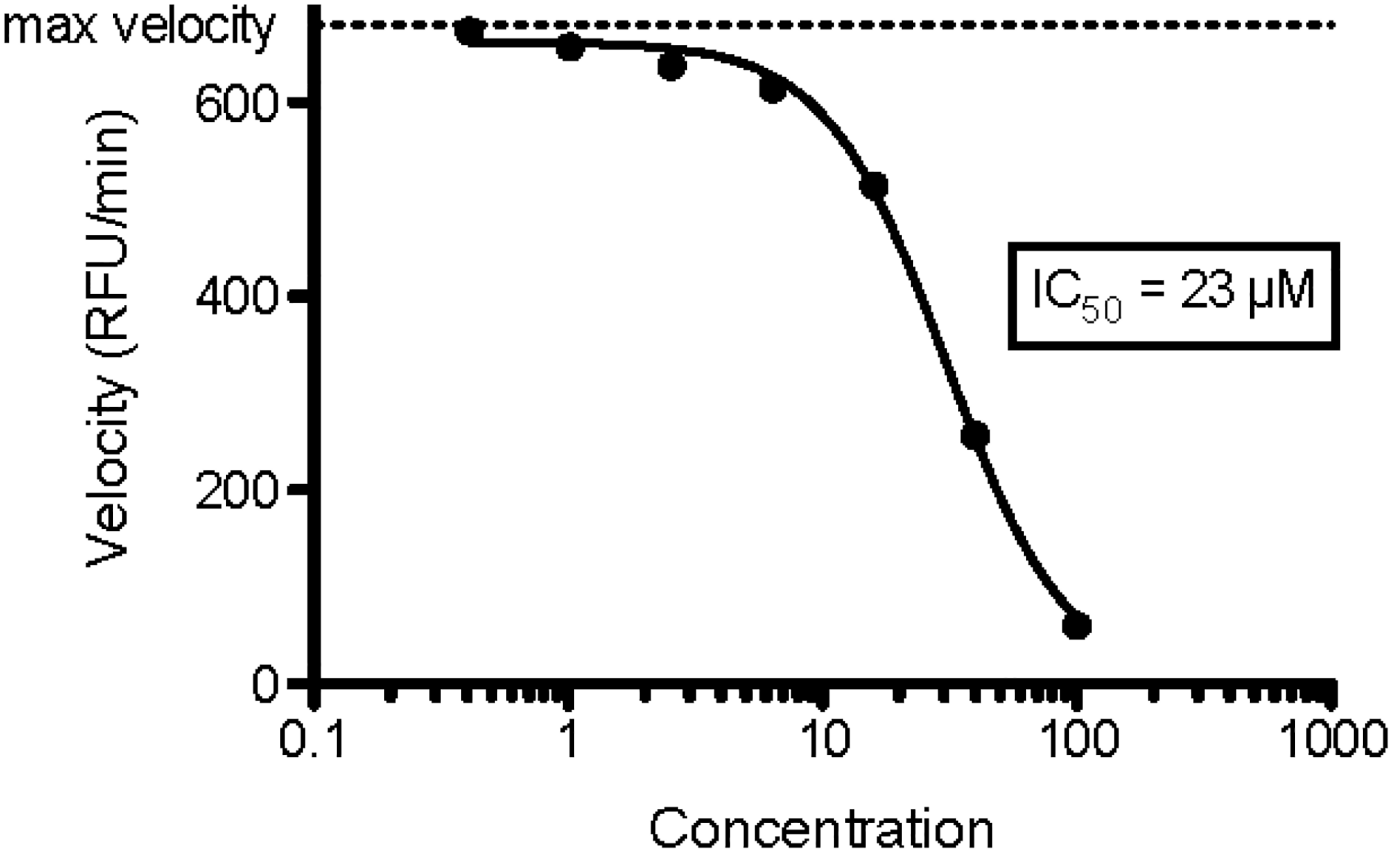
RecA data. RecA hydrolyzes ATP to ADP that can be enzymatically converted to H_2_O_2_, which oxidizes amplex red to the fluorescent molecule resorufin. The steady state rate of creation of the fluorescence is plotted on the y-axis in relative fluorescence units (RFU). The addition of a RecA inhibitor decreases the rate of production of RFUs in a concentration dependent manner. 6-Hydroxydopamine inhibits RecA with an IC_50_ of 23 uM. To our knowledge, this is an almost singular example of potently breaking a protein-protein interaction with such a small fragment and quite a remarkable prediction given how difficult it is to inhibit RecA (see Discussion section). The experiments were done in triplicate and all gave virtually the same binding affinity.

RecA forms complicated protein-protein, protein-nucleic acid and protein-ATP interactions. Fig 2 shows the results of SACP simulations on RecA with clustering and water exclusion. The first important thing to observe is that three binding sites are predicted (Fig 2a), which correspond to the three known RecA interaction sites. Detailed analysis of the predicted RecA-RecA Protein-Protein interaction site shows that the highest affinity fragment interactions occur at the universally conserved amino acids K216 and F217 (Fig 2b). All mutations of F217 dramatically reduce or eliminate the RecA-RecA interactions except for the F217Y mutant[86] that increases the protein-protein interaction by over two orders of magnitude indicating the importance of a single hydroxyl substitution at this position. The SACP simulations predict that phenol binds with higher affinity at this site in accord with the known experiments. SACP also predicts the new finding that benzene rings with multiple hydroxyl substituents bind in the position of F217 (Fig 2b) with even higher affinity than the mono-hydroxylated fragment and alkylamines bind in the position of K216 (Fig 2b) in a way that creates 6-hydroxydopamine. This prediction was so compelling that we decided to purchase 6-hydroxydopamine (6-HD) and perform the inhibition[87, 88] assay. As shown in Fig 3, 6-HD inhibits RecA with an IC_50_ of 23 μM. This is experimental validation of a notable and very surprising prediction, because 6-HD only weighs 166 Da. An interesting aspect of these results is that they do not rule out that 6-HD might be binding at the ATP-binding site of RecA instead of the predicted RecA-RecA interaction site. This possibility is unlikely, however, because many studies of kinases have demonstrated that 6-HD does not bind at the ATP site. A study of the ATP-binding purenergic receptor[89] P2X7, for example, indicates that 6-HD induces Parkinson’s like symptoms in an animal model in a P2X7 independent manner, which leads the investigators to conclude that 6-HD does not bind at the ATP site. What is also especially compelling about this finding is that the Singleton lab tested a variety of well-known ATP mimetics to inhibit RecA and all of them were ineffective, which is further explained in the Discussion Section.

### Hydroxamic acid fragments bind to and inhibit peptide deformylase through water mediated interactions

**Fig 4:**
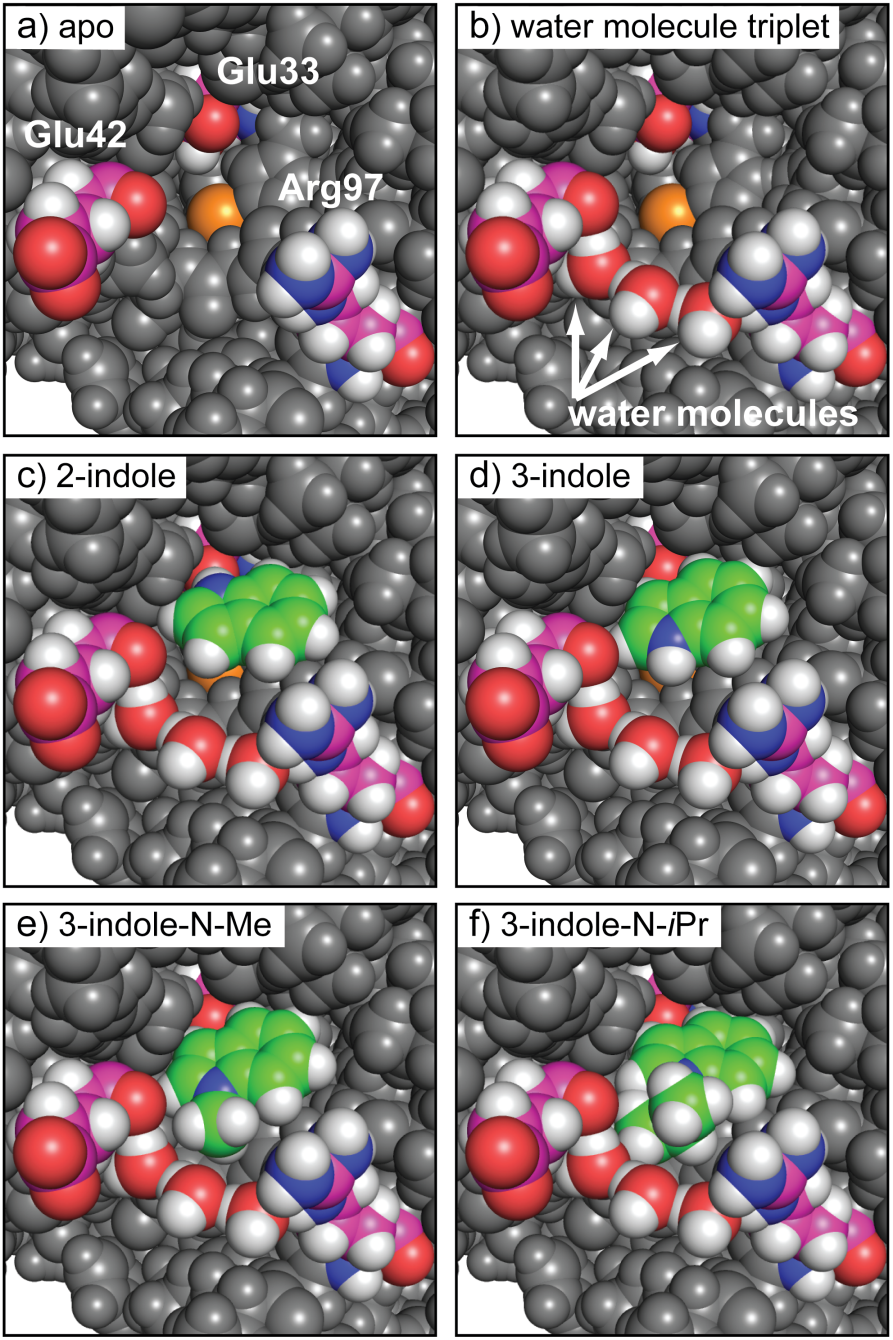
Binding poses on PDF. a) The PDF binding site consists of the buried Fe (orange) and Glu133 and the exposed Glu42 and Arg97. SACP predicts a water triplet (b) that bridges Glu42 and Arg97. SACP predicts that the 2-hydroxamic indole (C) binds deeply in the pocket H-bonding to Glu133 and has a higher affinity than the 3-substituted molecule, which is predicted to rotate out (d) of the pocket. SACP predicts that N-methylating the 3-sustituted compound (e) is destabilizing, because the methyl group is floating in a vacuum. The N-isopropyl compound fills the pocket (f) created by the waters and thus is predicted to have higher affinity.

**Fig 5:**
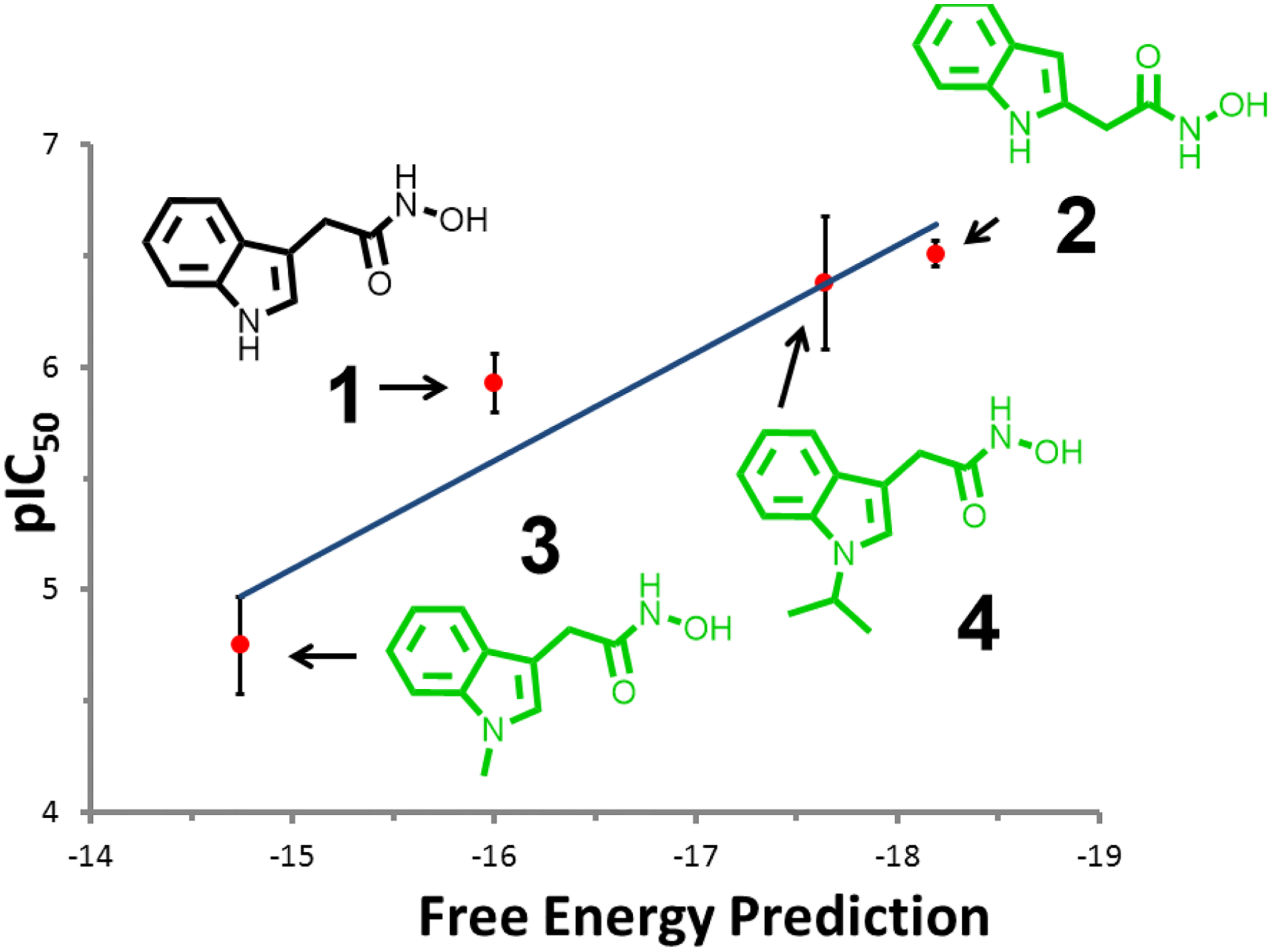
PDF data. The Boularot compound (**1**) is predicted to bind with fairly high affinity by SACP. The simulations predict that simply moving the acetyl-hydroxamic acid from the 3-position on the indole to the 2-position (**2**) will result in a more potent compound, which is confirmed by the experiments. SACP predicts that N-methylating this compound (**3**) will reduce the affinity, but that the N-isopropyl (**4**) derivative will increase the affinity, which is confirmed by the experiments.

N-formylmethionine is the universal amino acid used by bacteria to initiate[90] protein synthesis. Because the mature protein requires removal of the formyl group by the enzyme peptide-deformylase (PDF), small molecule inhibitors[65] designed to block this process are potentially a new class of antibiotics. The discovery that PDF is also active in mitochondria and that its inhibition can cause cells to enter apoptosis makes PDF an anticancer[66, 67] target as well. The binding of small molecules such as heterocyclic hydroxamic acids[91] to PDF are apparently complicated by water-mediated interactions[92] at the active site. Therefore, predicting and characterizing how fragments interact with PDF is especially challenging, due to the multi-body effects of bound waters. SACP simulations with clustering and water exclusion were applied to PDF with the results summarized in Fig 4. The PDF binding site (Fig 4a) is formed by the deeply buried Fe atom (orange) and Glu33 and the surface exposed residues Glu42 and Arg97. The simulations predict that a triplet of high affinity waters (Fig 4b) exists between Glu42 and Arg97, which are just outside of the binding site and effectively extend and close off the binding site. While we generally run about 100 fragments against any protein target, we focused the simulations and analysis on indoles carrying a hydroxamic acid functionality. We did this because Boularot[93] and covorkers, using NMR fragment-based screening, discovered that some of these derivatives are PDF inhibitors. Specifically, they found that indole substituted at the 3-position with acetyl-hydroxamic acid blocked PDF function with micromolar potency. We therefore used this molecule as a fragment in SACP simulations of PDF and we also created the 2-substituted molecule even though there are no reports to our knowledge on this compound. We usually create several different variations of each fragment, because in our experience it often results in surprising outcomes. In fact, this is exactly what happened; the previously unstudied 2-substituted indole is predicted by SACP to bind with higher affinity to PDF than the known 3-substituted indole. Higher affinity means that the 2-substituted fragment continues to bind at the site as the chemical potential is lowered, but the 3-substituted fragment has already disappeared. So as the simulation proceeds to lower chemical potentials there is a point where the 3-substituted molecule no longer binds to PDF but the 2-substituted compound still does and thus the 2-substituted molecule has a predicted higher binding affinity. We synthesized the 2-substitured molecule, and it does in fact have a higher potency than the 3-substituted molecule confirming the prediction. To discover the mechanistic reasons why this new 2-substituted compound has higher affinity than the reported 3-substituted molecule, we analyzed the SACP fragment data. Surprisingly, the 2-substituted molecule binds deeply (Fig 4c) into the PDF pocket making a hydrogen bond with Glu33. By contrast, the 3-substituted molecule (Fig 4d) cannot make this hydrogen bond—it is rotated 180 degrees and it binds near the protein surface and thus the interaction is somewhat weaker. SACP also makes another surprising and interesting prediction, N-methylating the 3-substituted molecule will destabilize the binding while the N-isopropyl derivative will enhance the affinity, which is seemingly very perplexing but reminiscent of hydrophobic[94] hydration. So we synthesized both molecules and in fact the assay data experimentally confirms the simulation predictions (Fig 5). The mechanistic explanation for these unexpected findings is that the methyl group does not fill the pocket created by the bound waters (Fig 4e) and hence it is partially floating in a vacuum, which is unstable. By contrast, the isopropyl moiety precisely fills the pocket (Fig 4f) formed by the waters and thus is predicted to be more stable, which is confirmed by the binding experiments.

To summarize the results of the SACP simulations, the 3-hydroxamic indole points the nitrogen of the indole towards the predicted bound waters. Another surprising result is the prediction that a propane fragment binds with a tight van der Waals interaction with these bound waters (Fig 4f) in a way that alkylates the nitrogen of the indole. Specifically, simply N-methylating this nitrogen is predicted to decrease the affinity of the 3-hydroxamic acid indole because it does not fill the cavity—the methyl group essentially sits in a vacuum and is thus destabilizing, but the n-isopropyl derivative is predicted to enhance the affinity because it does fill the cavity with the right van der Waals contacts. These molecules were synthesized and assayed, which confirm the computational predictions of the relative rank ordering of these four compounds as shown in the graph displayed in Fig 4. Trial and error synthetic expansion on the nitrogen might possibly have found these derivatives, but SACP predictions narrow down the variations and focuses on the most likely improvements. This set of results shows how complicated fragment-protein interactions can be when water mediated interactions must also be accounted for. Thus, using SACP simulations to first locate the tightly bound waters and then using these waters in subsequent SACP simulations to discover their influence on how organic fragments bind is a powerful way of interrogating the multibody protein-fragment-water architecture.

### Accurate water mapping is essential for characterizing the binding site of HIV protease

We have found that in almost all cases protein binding sites and sites of high affinity protein-protein interaction have very low affinity for water and thus the water exclusion principle is an essential part of our hypothesis on molecular recognition. HIV protease is an important exception, because this enzyme has one essential molecule of water at the center of its binding site. The co-crystal structure of HIV protease[49] with one of the earliest drug-like inhibitors[95] crixivan, immediately established the central role of water as a key mediator of ligand binding in the active site. Numerous recent studies[96–99] have demonstrated that resistance mutations to protease inhibitors is critically dependent on the hydration state of the active site, and thus water binding must be taken into account in the development of any new inhibitors. Early efforts to understand the mechanism of HIV protease led to novel protein constructs that enabled high resolution crystal structures[100, 101] of the Apo protein. All of these efforts revealed a key structural water molecule that bound to both catalytic aspartates of the HIV protease dimer. Thus, a principled way of understanding and predicting the characteristic hydration of HIV protease is essential for understanding its fundamental molecular recognition processes.

**Fig 6:**
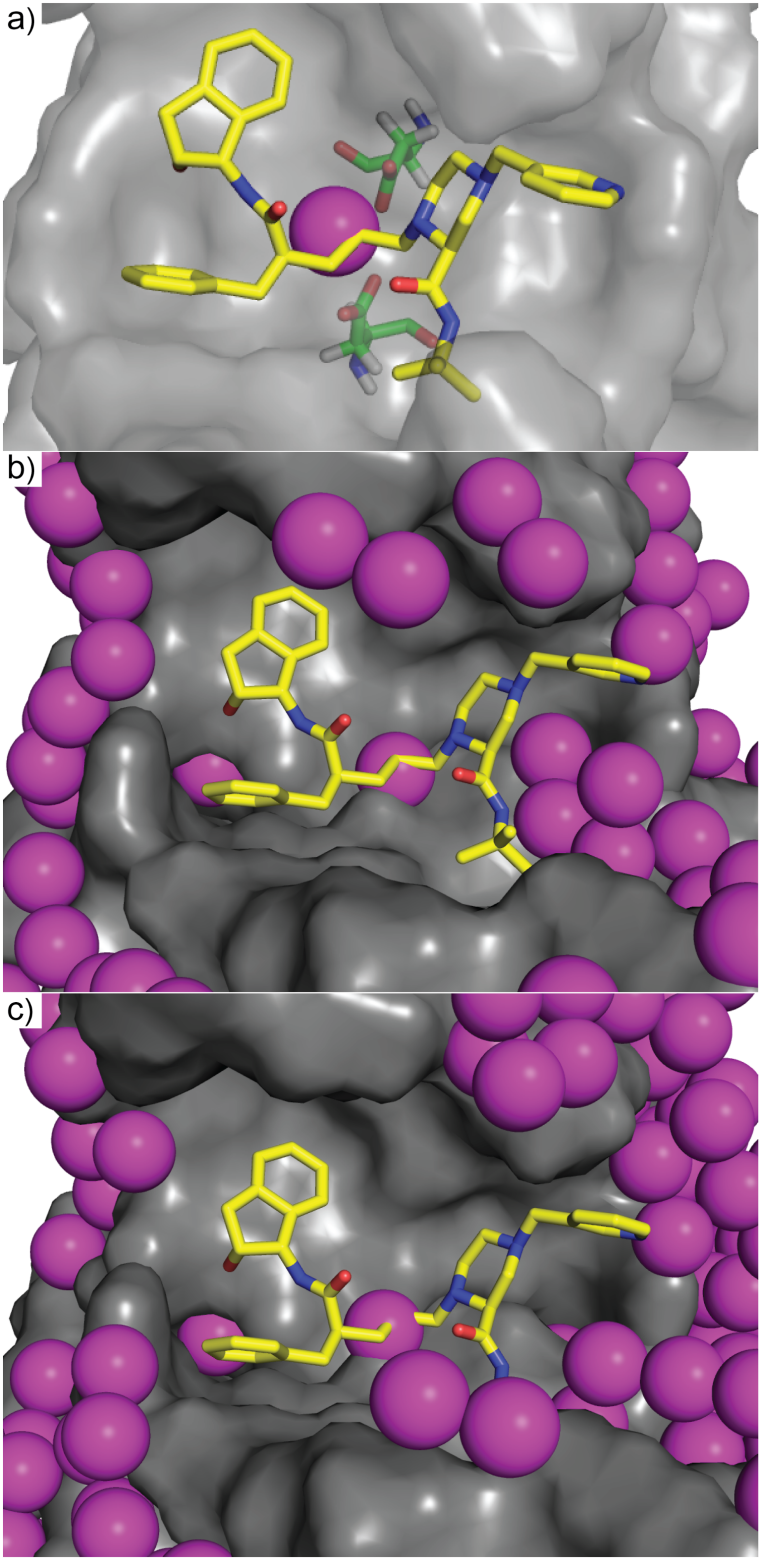
Water data on HIV protease. The SACP water molecules predicted to bind to HIV protease. The simulation produces that key water molecule (a) experimentally known to be at the center of the binding site. All of the other water molecules from SACP obey the water exclusion principle and bind in a ring just outside of the site (b-c). Two key aspartates are at the center of the binding site (Asp25 and Asp125 colored with green carbons in panel a). The ligand indinavir is shown with yellow carbons in all three panels. Part of the protein surface is hidden to reveal the binding site. Three different protonation states were simulated, a) ASP25 and ASP125, b) ASH25 and ASP125, c) ASH25 and ASH125 with all giving comparable results.

**Fig 7:**
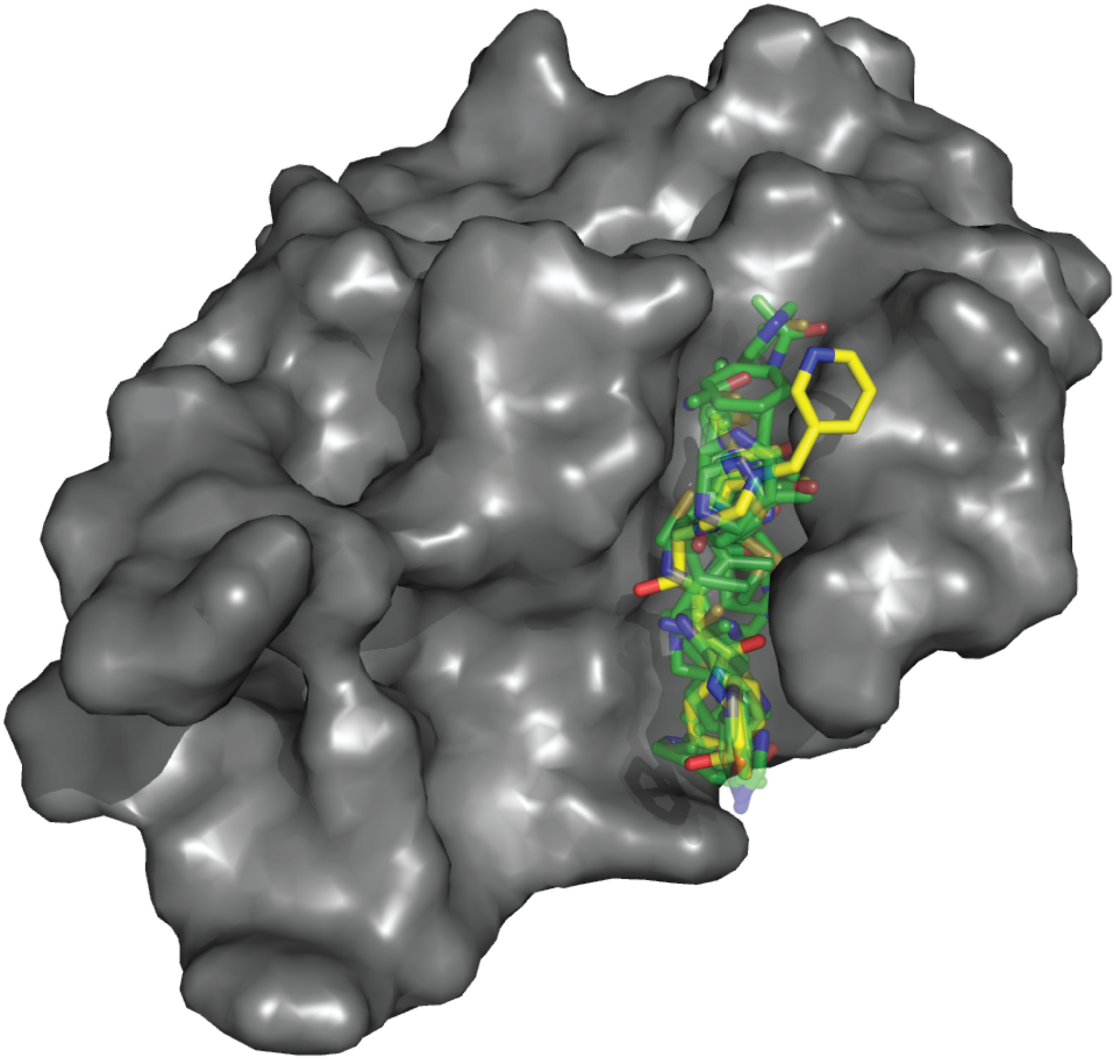
Hot spot on HIV protease. High affinity diverse fragment clustering completely maps out the indinavir binding site on HIV protease.

An important factor in the study of HIV protease is the protonation states[102] of the catalytic aspartates, so SACP of water was run on the structure (1SDT) with both aspartates deprotonated, with one protonated and the other deprotonated, and with both protonated. In all cases SACP finds one isolated water molecule at the center of the binding site at very low chemical potentials indicating that it has very high affinity for the protein at this site. What is very interesting is that HIV protease, with this one singular exception, otherwise obeys the water exclusion principle. Fig 6a shows the SACP predicted water binding to both catalytic aspartates in the exact position found in the crystal structures with this water present even at a chemical potential of -29 in the singly deprotonated state and -25 in the doubly deprotonated state. Paradoxically, it might be expected that have both aspartic acid residues (ASPs) negatively charged would have a higher affinity for water, but SACP in accord with both experiments[103] and computations[102] indicates that the pKa for this site means that one ASP is protonated and one is deprotonated. As the chemical potential is increased by approximately 20 units to -8 for the singly protonated state and -5 for the doubly protonated state low affinity water molecules fill in the boundaries of the binding site but no new additional water molecules are found in the binding site itself (Fig 6b and 6c). Even in this case over a large range of chemical potential values SACP shows that the water exclusion principle is obeyed by HIV protease with one functional exception. Finally, applying the principle of high affinity fragment clustering, the HIV protease binding site is readily mapped out as shown in Fig 7.

To summarize these results, Fig 6a shows that the singular water molecule at the HIV active site identified by X-ray crystallography is identical to the water molecule predicted by SACP simulations. Additionally SACP also predicts that the lower affinity bound waters define the boundaries of the binding site, which obeys the water exclusion principle that binding sites are generally devoid of very high affinity waters. Thus, the key water at the center of this binding site is predicted by SACP to be an exception to the water exclusion principle. Finally, in Fig 7 we show clustered organic fragment binding data superimposed[95] over indinavir, illustrating that SACP with no free parameter adjustments actually recreates known ligand binding patterns.

### Locating the point of electron transfer in the large extensive binding site of dihydrofolate reductase

**Fig 8:**
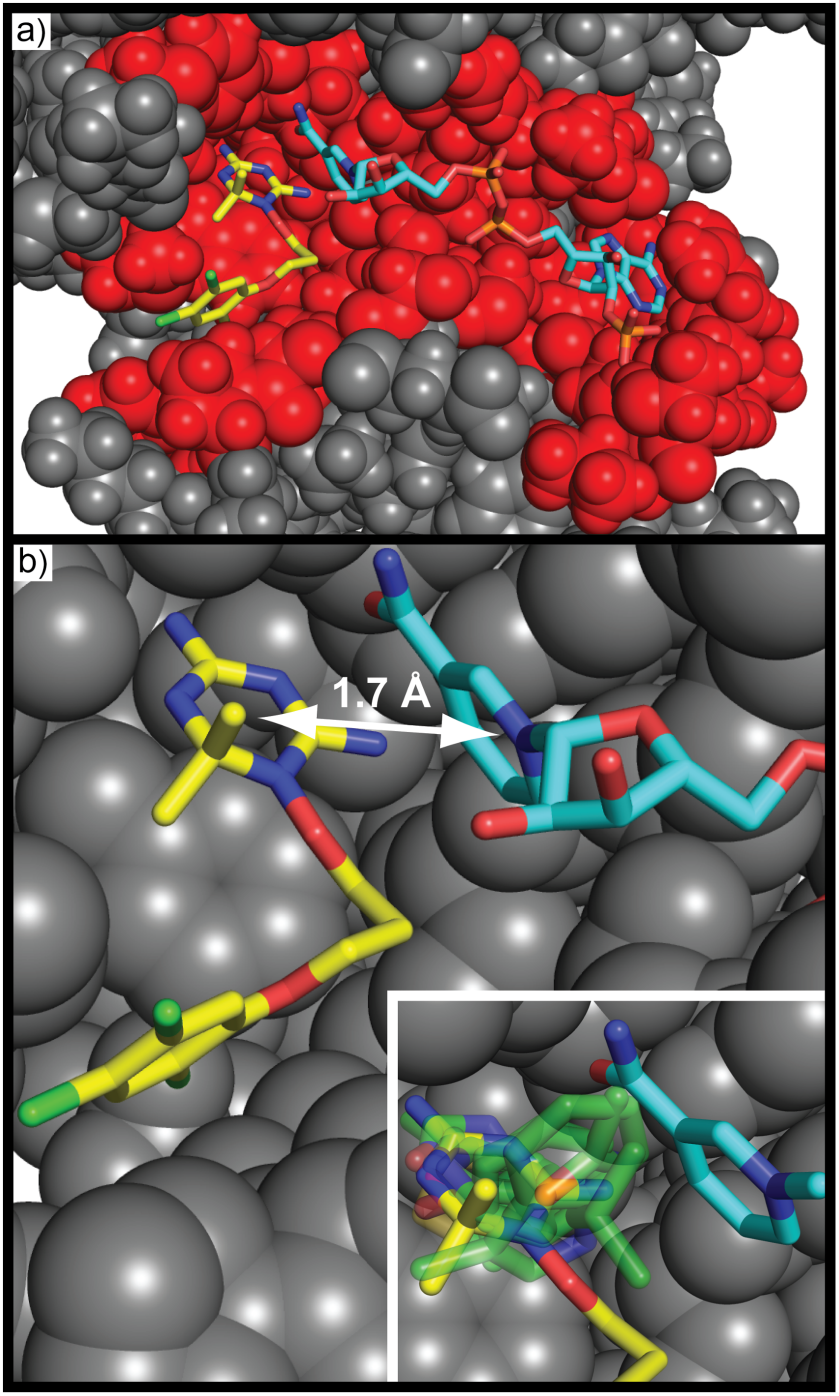
Hot spot on DHFR. DHFR complexed with WR99210 (yellow carbon atoms) and NADPH (cyan carbon atoms) is shown. Some residues are hidden to reveal the binding site. DHFR has an extensive (a) binding site; all residues within 4 Å of ligand and cofactor displayed with red spheres. Two aromatic rings (one on WR99210 and another on NADPH) are separated (b) by ~1.7 Å—this is an impossibly high energetic configuration under normal circumstances, which the protein sets up. SACP (inset: fragment cluster & water exclusion presented with partially transparent green carbon atoms) predicts a high affinity pinpoint site between these rings showing that the extensive binding site is scaffolding that creates this functional site.

Dihydrofolate reductase is an enzyme that catalyzes the reduction of folic acid by carrying out an electron transfer from the cofactor NADP. A simple binding site analysis of wild-type Plasmodium falciparum dihydrofolate reductase-thymidylate synthase (PfDHFR-TS) complexed with WR99210 –displaying the residues that are < 4 Å from any ligand atom shown in red in Fig 8a—clearly shows that the two ligands contact a large and extended binding site of the protein. A more detailed analysis shown in Fig 8b reveals that one aromatic ring from each ligand, although interacting noncovalently, are only separated by 1.7 Å. These nonbonded rings are placed within almost covalent distance, because this is the region where electron transfer occurs. SACP simulations strikingly predicts the highest affinity cluster with water exclusion lies exactly between these two rings as shown in the inset of Fig 8. To summarize these results, although DHFR apparently has a very large extensive binding site, the electron transfer function occurs at a point between two aromatic rings separated by only 1.7 Å–an impossibly high energy state that can only occur within this type of binding site. SACP with clustering and water exclusion easily locates this singular functional hot spot as the one key site of this protein.

### Experimental and computational fragment-based studies locate the binding subsites in elastase

Elastase has been a historically important protein[104, 105] since 1969, with the discovery that its hyperactivity is the central mechanistic cause[106, 107] of emphysema. The first co-crystal structure of elastase bound with a substrate[43] was published in 1976 by Alber, Petsko, and Tsenoglou, but the first publicly available structure deposited in the PDB in 1986 was solved in 1982 by Hughes[44] and coworkers. The elastase co-crystals solved by Meyer[45] and colleagues and the subsequent inhibitor-elastase high resolution structure solved by Navia[46] and co-workers showed the ligands in the same configuration as the Hughes structure confirming comparable binding modes. Petsko, 18 years after solving the first co-crystal, demonstrated with his colleague Ringe that in fact almost identical substrates could bind in radically different configurations, thus discovering new binding sub-sites in elastase[47] almost 2 decades after the first structure was solved.

**Fig 9:**
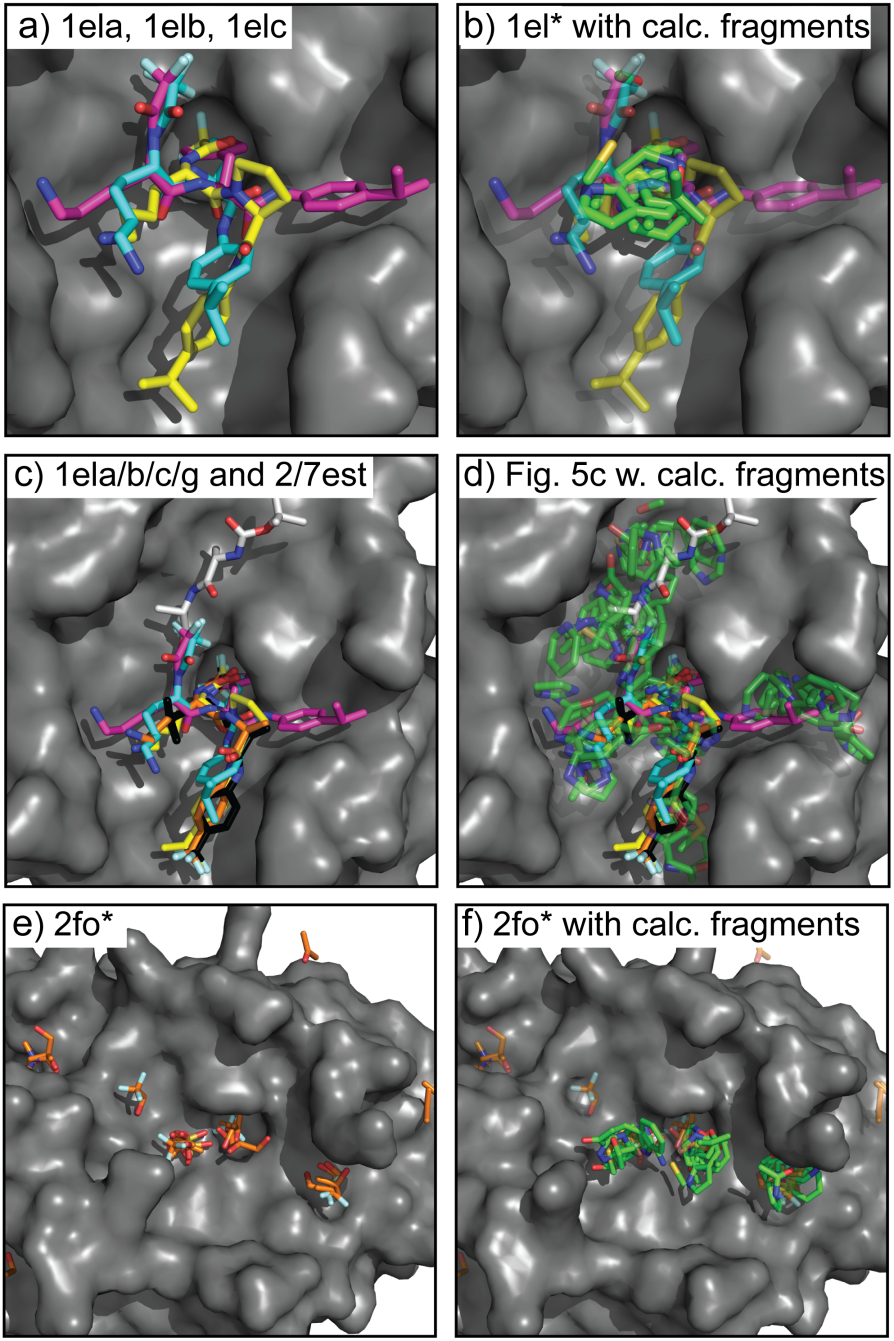
Hot spots on elastase. Overlay of 3 different (a) elastase co-crystal structures 1ela (yellow), 1elb (cyan), and 1elc (magenta). Beginning in 1976 and continuing until 1994 all of the elastase co-crystals had the ligand binding in the same mode as 1ela. The 1elb and 1elc structures solved in 1994 revealed new binding subsites. The highest affinity (b) SACP fragment clusters (green carbons) with water exclusion are at the site where the 3 ligands overlap. This result is the same for simulations using the 1ela, 1elb, and 1elc structures. Nine co-crystal (c) structures (2fo9, 2foa, 2fob, 2foc, 2fod, 2foe, 2fof, 2fog, 2foh) are needed to map out the entire binding site in elastase. SACP fragment binding predictions using the top three highest interactions (d) cover the subsites from all 9 co-crystals, indicating that these sites were long undiscovered because that have lower affinity for the ligands. Importantly, experimental X-ray fragment-based soaking experiments (e) done in the Ringe Lab did reveal the lower affinity hard-to-find subsites. Using this crystal structure as input into SACP, the simulations (f) also find all of the subsites.

The long progression of co-crystal structures required to uncover hidden binding subsites in elastase demonstrates some of the challenges and mysteries of molecular recognition, which makes it an excellent test case for fragment-based molecular recognition studies. The results of SACP simulations with clustering and water exclusion are summarized in Fig 9. Three peptide co-crystals with the ligands superimposed (9a) show experimentally that there is a tightly focused hot-spot where all three ligands overlap. Typically, we perform a preliminary initial set of SACP calculations on 30 organic fragments and water. Recall, that these 31 simulations are performed simultaneously and independently on different CPU processors to discover the free energy of binding to the protein in all cases separately, so different fragments have no knowledge of each other. Also recall, that for a given simulation the fragment-fragment self-energy is part of the simulation, because the protein is completely solvated by this organic fragment and annealing of the chemical potential goes through a phase transition that evacuates the “solvent” leaving behind tightly bound fragments. This is identical to the fragment soaking experiments done with experimental X-ray crystallographic methods. In our previous communication on hen egg white lysozyme, we present SACP results using acetonitrile as a fragment and the post phase transition of the dozen fragment binding positions matches the X-ray studies done in the Ringe Lab.

Typically, after all of the individual fragment simulations are complete the clustering algorithm processes the data and discovers where 10 diverse fragments bind to the protein within 2A or less of each other. In the case of HIV protease as shown in Fig 7, the fragments cover the extensive binding site exactly where crixivan is located. This happens, because each fragment is slightly offset from a different fragment and thus a chain across the whole binding site is formed. In elastase, by contrast, the fragments all pile up in a tight location (Fig 8b) right at the place where the 3 different ligands intersect (Fig 8a). This localized region is discovered by SACP when only the highest affinity (lowest chemical potential) binding fragments are used for the clustering algorithm and as always water exclusion is applied so that we know that water does not bind tightly at this site. Water exclusion using explicit water binding with SACP is a rigorous way of knowing whether or not organic fragments can effectively bind, because water does not bind tightly at this location.

Fig 9c shows a superimposition of 6 different co-crystal structures—the same three from Fig 9a and three more solved by Petsko and Ringe. The important thing to note is that one of the new co-crystals binds in the same mode as the older 3 structures so it provides no new information, but the two others bind in totally different modes defining previously unidentified sub-sites. The method for fragment clustering involves several parameters: (i) the number of fragment types used (chemical diversity), (ii) a radius for fragment cluster assignment, and (iii) the predicted affinity of the fragments. To fully map out the binding sites of the elastase peptides, we included the second highest affinity binding fragments, increased the clustering radius from 2 to 3 Å, and lowered the chemical diversity from 10 fragments down to 9. By doing this, all the new subsites from the 6 agglomerated co-crystals were identified (Fig 9d).

A different way of doing a comparable analysis of elastase binding subsites is to crystalize the protein in the presence of different small organic molecules, a process known as multiple solvent crystal structures (MSCS). The protein is crystallized in the presence of one fragment acting as the solvent. This is done again with a single different fragment and then repeated with many fragments individually. The protein is not crystallized in the presence of multiple fragments simultaneously. High resolution X-ray structures are obtained in each case, which always reveals that each fragment has a unique spectrum of binding sites spread out over the entire surface of the protein. Locating the sites on the protein that have a high affinity for a diversity of fragments invariably reveals the binding site. Mattos and coworkers mapped the binding surface of elastase with this technique to find all of the local hot spots (Fig 9e).[108] MSCS is a form of fragment-based X-ray crystallography that successfully mapped out all of the binding sub-sites of elastase. The ease of finding all of the elastase binding sub-sites with theoretical or experimental fragment-based methods such a SACP or MSCS, respectively, may be contrasted with the difficulty of identifying them with experimental co-crystallography, because not only did it take almost two decades, but Petsko and Ringe had to solve fifteen different co-crystals before all of the hot-spots were experimentally located and characterized. SACP identified these sites (Fig 9f) using only one crystal structure. The definition of the hot spots, using chemical diversity and chemical radius, with clustering and water exclusion in order to find all hot spots demonstrates these principles can be used to identify and characterize the distribution of multiple binding modes in elastase and the singular functional site in DHFR.

### MDM2-MDMX-p53—Identifying the subtle differences in homologous protein-protein interactions

**Fig 10:**
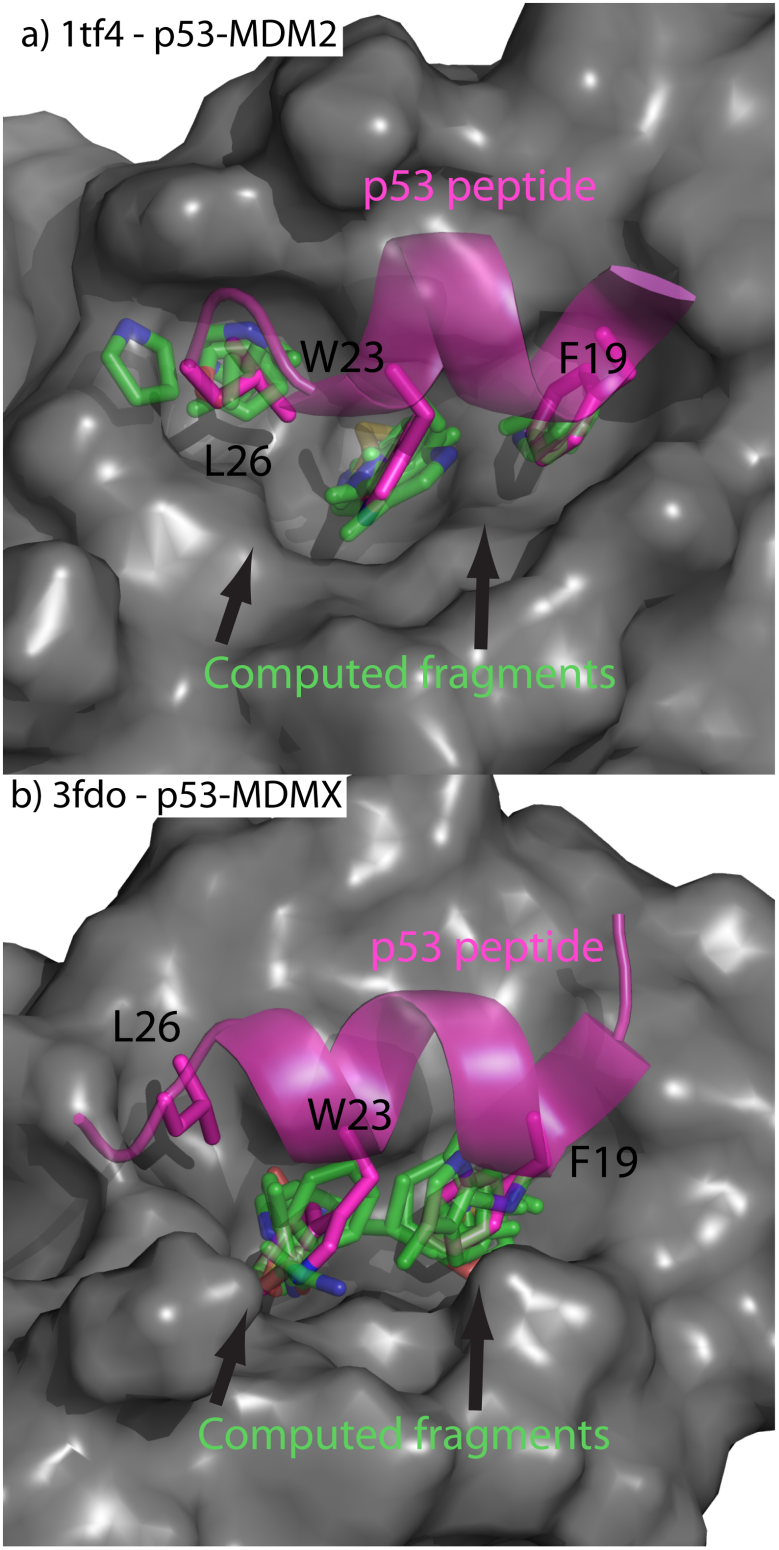
Hot spots on MDM2/X. a) p53-MDM2 protein-protein interaction displayed with overlay of computed fragments. b) p53-MDMX protein-protein interaction displayed with overlay of computed fragments. The MDM surfaces are presented in gray. The p53 peptide is colored magenta. The computed fragments are shown in green. Strikingly, the fragments predict a high affinity site at L26 for MDM2 but not MDMX, which is in accord with the experimental finding on the differential binding.

Protein-protein interactions (PPI) typically have large surface area and appear[109] featureless, which hinders the development of small molecule inhibitors. A great challenge in the PPI of cancer is to identify the subtle differences between how MDM2 and MDM4 bind to p53. From previous computational[110] and experimental[52] studies it has been discovered that the three most important residues in this PPI are F19, W23, and L26 (Fig 10a and 10b). SACP Hot-spot identification using clustering with a 2A fragment radius and water exclusion on MDM2 and MDM4 identified the binding site at W23 indicating that this is the highest affinity interaction point. Increasing the cluster radius to 3 Å with lower fragment diversity returns a second cluster that binds at F19 on both MDM2 (Fig 10a) and MDM4 (Fig 10b). Interestingly, these two clusters are tighter and better focused in MDM2 than in MDM4. If we continue to lower the restraints by making the cluster radius 4 Å and asking for less diversity, there is a binding hot-spot at L26 on MDM2 (Fig 10a) that does not exist for MDM4. To discover the reason for this subtle difference, we analyzed the binding clusters further and discovered that MDM4 has a methionine at position 54 that impinges on the pocket around L26, whereas by comparison[54] MDM2 has a corresponding leucine in a comparable spatial position at residue 53 that does not impinge on the L26 pocket. Another important difference between the structures of MDM2 and MDM4 is the presence of a second hydrophobic binding site, adjacent to the L26 site, in MDM4—formed by L33, V52, and L106 and separated from the P53 binding site by M53.

Further reducing the restraints of the hot-spot algorithm, the second hydrophobic site is revealed on MDM4 but no site is identified on MDM2. This site could be utilized to discover novel, and selective, MDM4 inhibitors. SACP hot-spot analysis not only found clusters over the three most critical residues, but also ranked these clusters, which is in agreement with a computational alanine screen[110] and an experimental alanine screen.[52] By rank-ordering the fragments by affinity, using a diverse fragment set, and excluding sites that have waters, we locate the sites of high interaction energy and can rank those sites and thus uncover the differential binding to p53 of these 2 highly analogous proteins.

### Understanding the HIV TAT-TAR protein-nucleic acid interactions requires locating and characterizing the binding sites on the viral RNA

**Fig 11:**
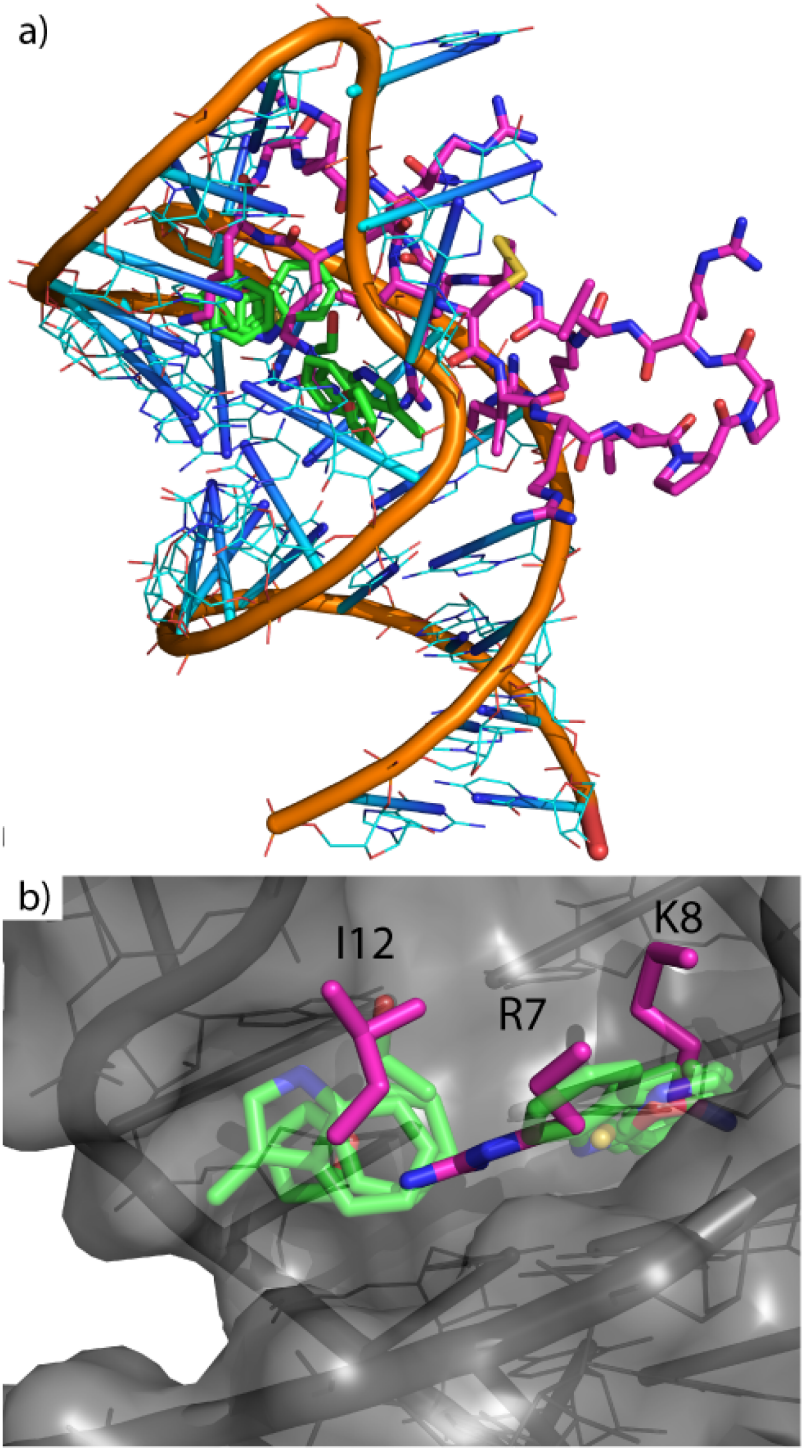
Hot spots on HIV TAR. a) HIV RNA (orange backbone with cyan nucleosides) and TAT peptide (magenta carbons) with calculated clusters (transparent green carbons). b) Zoomed in and displays overlap of two clusters on residues I12, R7, and K8, which are the experimentally known key interactions.

To further demonstrate the generality of the SACP system for identifying and characterizing key sites of molecular recognition, the simulations were run on the HIV TAR RNA stem loop. RNA offers a unique system for hot-spot mapping because RNA can fold into unique 3-dimensional structures, as proteins do, but RNA is only made up of four different bases, which can appear chemically uniform. TAR RNA is found at the end of all viral transcripts, and cyclic peptides that inhibit this protein-RNA interaction have been shown to slow viral replication in all clades[59] tested. The cyclic peptides clearly indicate a small set of neighboring highly localized protein-RNA molecular recognition sites. I12, R7, and K8 are the three critical residues required for peptides mimicking the native HIV binding protein, TAT. Cluster analysis with water exclusion predicts two neighboring sites of molecular recognition; one cluster overlapping K7 and part of R8 with another cluster over I12 and part of K7 (Fig 11). Even though RNA is chemically uniform, hot-spot mapping with SACP was able to reveal the binding sites on TAR RNA that are associated with the key residues on the Tat mimetic in the 2kx5 conformation. The only input to the simulation is the RNA structure and the fragments including water. Post phase transition cluster analysis and water exclusion immediately produce the site of RNA-protein interaction where known peptide inhibitors bind.

## Discussion

Four crucial aspects of these studies are, a) the sampling technique that will be illustrated by the multibody water-water-protein interactions, b) generating a phase transition to determine locations on the protein that have high affinity for a fragment, c) interpreting the data in order to predict new experiments, and d) the need for complete and accurate water mapping over the whole protein surface.

### Predicting the well-known experimental multi-body water triplet in BPTI illustrates SACP sampling

**Fig 12:**
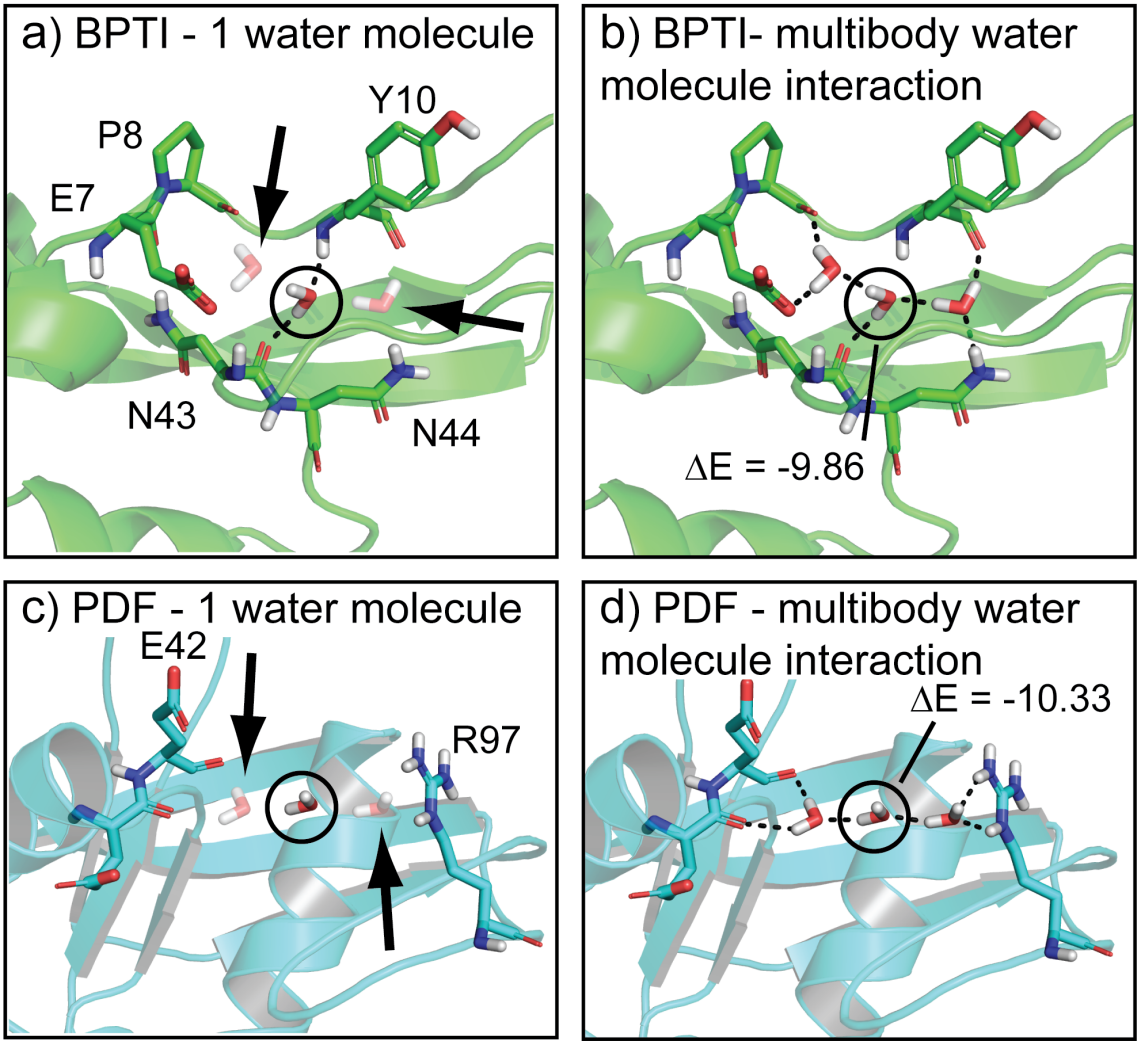
Multi-body water interactions. a) The water triplet of BPTI is shown with the central water circled and its neighbors faded (arrows) to indicate that we performed an energy calculation on the middle water with the others not considered. When all the waters are included (b) the energy of the central water drops by approximately 10 kcal/mol. Annealing the chemical potential with SACP simulations correctly predicts the BPTI water triplet, because the unstable water is acceptable at high chemical potentials. Continued sampling at high chemical potential enables discovery of the other water molecules and creation of the highly stable multi-body triplet that survives driving the system through the phase transition. SACP predicts a similar water triplet in PDF. Like BPTI, the central water has a high energy when the other two waters are not considered (c), but when all three waters are taken into account (d) the energy of the central water drops by more than 10 kcal/mol.

The bovine pancreatic trypsin inhibitor (BPTI) has been studied extensively with X-ray[111–115] crystallography, NMR[116], and neutron[117] diffraction. The most striking motif of BPTI is a deeply buried water triplet that is completely enclosed by the protein. There is no way to discover this water-water-protein multi-body triplet with molecular dynamics or canonical Monte Carlo, because both techniques require that water diffuse through the protein—one method driven by Newtonian forces and the other with a random walk. In principle, grand canonical Monte Carlo can discover these deeply buried waters, because the process can insert and delete new water molecules into cavities, but it does not (we ran many such simulations on BPTI and it never produced the water triplet), because it maintains a constant chemical potential. SACP, however, does find the water triplet and is the only method in our hands capable of doing so. The reasons for this are illustrated in Fig 12. During a grand canonical simulation, any particular water inserted into this cavity has a very high unstable energy, because it is at least partially flanked by a vacuum and is therefore rejected as a probable configuration. At the elevated chemical potentials of the early phase of SACP simulations that overcome high energy barriers, however, one water molecule has a dramatically enhanced probability of being an acceptable configuration. Subsequently, when additional trial waters are inserted in the presence of this first isolated water, the collective water-water-protein interactions are so stable with such a dramatic drop in energy the state is immediately accepted. Because this is a very low free energy state, a high fraction of the randomly generated replicates subject to deletion steps fail to break up these collective interactions and thus this water triplet remains intact even as the SACP simulations go through the phase transition and proceed to very low values of the chemical potential. Obtaining the free energy of protein hydration and dehydration is essential for accurate molecular recognition predictions and SACP is the only method to our knowledge capable of doing this.

In almost all cases that we have studied, binding sites do not have tightly bound water molecules—going through the phase transition depopulates water from these regions of the protein. As pointed out in the results section, HIV protease is an exceptional case with one tightly bound water molecule at the center of the binding site. PDF turned out the be another unusual case—SACP produced a water triplet (Fig 3c and 3d) at the very edge of the binding site that survives driving the simulation through the phase transition. This is reminiscent of the triplet in BPTI—the multibody interactions are essential for the stability of this configuration. These very low free energy interactions combined with the compelling way that the edge of the binding site is closed and completed, motivated us to keep these waters in the analysis of other fragment simulations and use the predictions to carry out new binding experiments. These results, which could in principle be observed with experimental fragment-based techniques, can only be obtained computationally with SACP to the best of our knowledge, because it is the only method in our hands capable of doing the extensive sampling required.

### The phase transition

All multi-body fragment-fragment interactions are taken into account, which forms a collective network of solvent-solvent interactions around the protein. A phase transition is generated by successively lowering the chemical potential until a free energy capable of breaking apart the solvent-solvent (fragment-fragment) interactions is discovered and suddenly fragments are rapidly deleted from the simulation cell. At this point, fragment-protein interactions are effectively in competition with fragment-fragment interactions. Going through the phase transition, a fragment will only continue to bind at a specific location on the protein if this fragment-protein interaction has a higher affinity than the fragment-fragment collective interactions.

To be clear, at high chemical potentials, the chemical potential free energy dominates the simulations and fragment insertions have a high probability, which fills the entire simulation cell and every cavity in the protein large enough to accommodate a fragment. As the chemical potential is lowered, very little happens until this chemical potential approaches the free energy of the multibody bulk fragment-fragment interactions. When the chemical potential is lowered beyond this point, these bulk interactions are broken essentially destroying the global solvent cohesion and fragment deletion becomes highly probable and the simulation cell becomes rapidly depopulated of fragments. Fragments that have an interaction free energy with the protein that is much greater than the fragment-fragment interactions survive driving through the phase transition. This process discovers the sites on a protein that have higher affinity for a fragment than a fragment has for itself. Experimental fragment-based studies bathe the protein in a solution of a particular fragment and then crystallize this protein to see where fragments stick or use NMR to determine where they bind. The SACP process mimics these fragment soaking experiments in a rigorous way.

### Designing experiments

**Fig 13:**
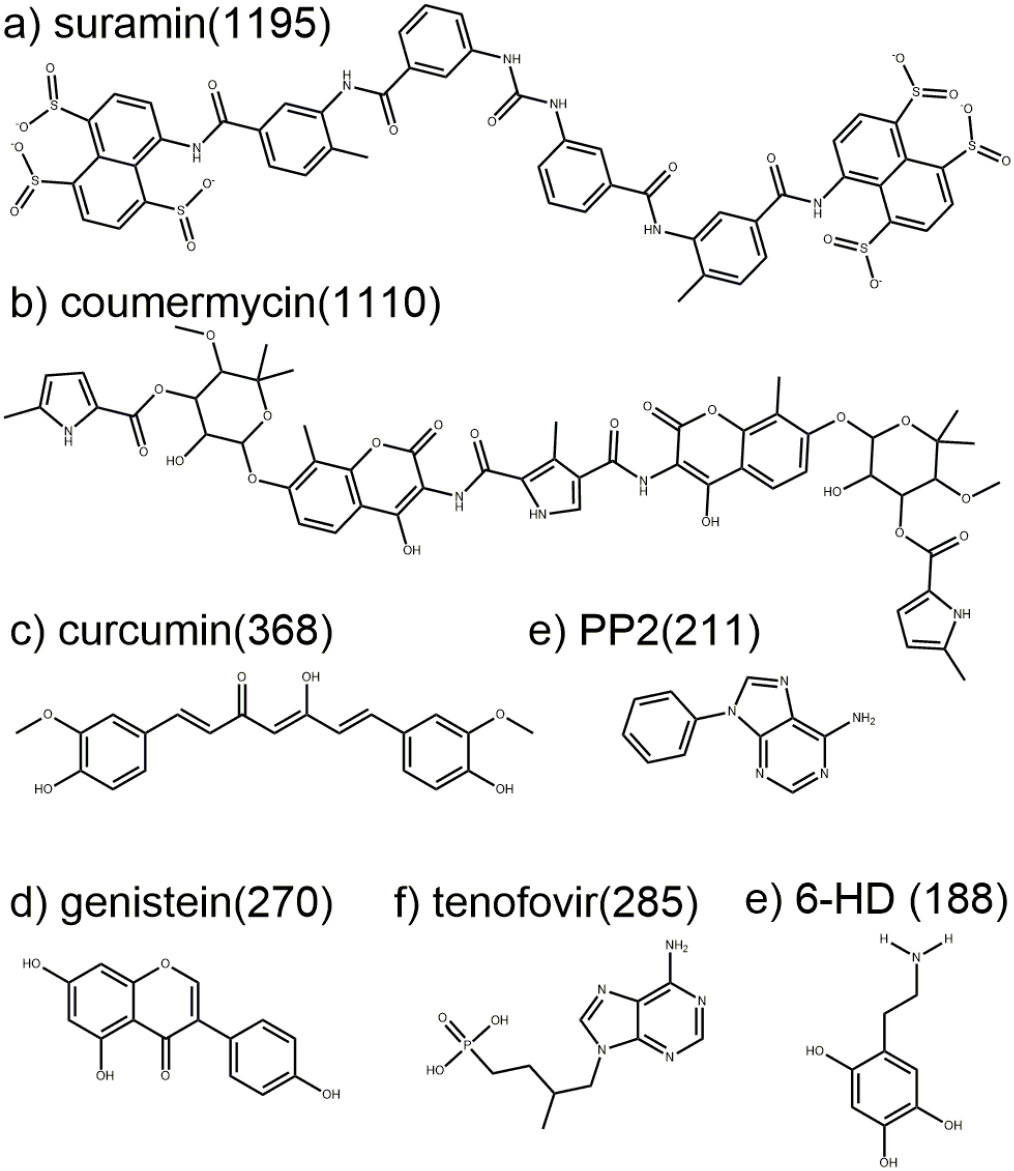
RecA ATPase inhibitors. Many different classes of ATPase inhibitors were tested on RecA and shown to have no effect. a) Suramin was developed in the early 1900s to treat parasitic diseases like African sleeping sickness and is on the World Health Organization’s list of essential medicines. b) Coumemycin is a natural product produced by Streptomyces, which potently inhibits staphylococcus gyrase. c) Curcumin and d) genistein are natural products that have been widely studied as antibiotics and anticancer agents. e) PP2 inhibits tyrosine kinases and has been widely studied as an anticancer agent. f) Tenofovir is an FDA-approved treatment for human immunodeficiency virus 1 (HIV-1) and hepatitis B virus infections. e) 6-HD discovered with SACP is the only selective inhibitor of RecA within this group. Numbers in parentheses are molecular weight for each compound.

It is highly nontrivial to construct new experiments to test simulation predictions, because there is a great deal of data produced by SACP that needs to be analyzed. One convenient starting point is to examine how well the simulations reproduce what is already known. With regard to RecA, the protein-protein interaction site contains several amino acids that are universally conserved in all bacteria, with the most important ones being a phenylalanine and a lysine. The SACP simulations not only reproduced these key interactions, but also predicted that tyrosine has a higher affinity than phenylalanine, which RecA investigators have experimentally demonstrated with mutagenesis experiments. Totally new findings from the simulations were that multiply hydroxylated benzene rings are predicted to have even higher affinity, and that alkylamines could overlay the lysine side chain with the correct geometric bond lengths and angles for binding to the benzene ring. This analysis resulted in the prediction that 6-hydroxydopamine would have high affinity for the RecA-RecA protein-protein interaction site. A review of the RecA literature[87, 88, 118–121] indicates that all screening efforts have failed to produce any molecule that inhibits RecA including a very wide variety of historically important ATP mimetic inhibitors (Fig 13) and thus finding anything to block RecA activity would be an extremely difficult and unprecedented challenge. Nevertheless, the SACP results were so compelling that we decided to have 6-HD assayed, and as described in the Results section it is very potent for such a low molecular weight entity.

Knowledge of alternative binding modes provides new and different avenues for optimization in drug discovery. When SACP uncovered that different substitution patterns of hydroxamic acids can bind in different orientations to PDF, this compelled us to synthesize a new derivative and perform binding experiments. When the experiments confirmed the hard-to-explain SACP predictions, we dug a little deeper into a possible mechanistic reason for the findings. This led to an even more surprising result that bound waters play a crucial role for some derivatives. Before synthesizing any more compounds, we checked on the validity of the bound triplet water predictions for PDF by simulating a very well-known protein, BPTI. When the simulations accurately reproduced the known water triplet deeply buried in BPTI, we had enough confidence to trust the new predictions on PDF and to synthesize a couple of compounds to test the water-mediated binding predictions. The binding experiments confirmed the computational predictions. It is a difficult decision to commit experimental resources using only the results of a simulation, so we will often run closely related test cases whenever possible. Success on the test cases provides a measure of confidence that motivates doing the experiments.

### The crucial nature of water binding

Water plays a special and complicated role in molecular recognition. It is absolutely essential to determine the sites on a macromolecule that have high affinity for water. Dehydrating a protein or nucleic acid by driving it through a phase transition quantitatively determines the details of water binding at every site of the macromolecule. When the system approaches a chemical potential that enters the phase transition, it is at a free energy that begins to break the water-water-water solvent cohesion. Thus, an additional very small lowering of the chemical potential completely destroys the multi-body solvent cohesion and all bulk water molecules are evacuated from the system. Many of the waters bound to the macromolecule are also removed during the phase transition, but a few finite sites on the macromolecular continue to have bound waters often in a multi-body configuration after the phase transition. What does this mean physically? The sites on the macromolecule that are unable to continue holding on to the water molecules through the phase transition indicate that these sites have lower affinity for water than the bulk water-water-water interactions. By extension, this means that at these sites the bound waters rapidly and spontaneously exchange with the bulk. The sites on the macromolecule that retain water binding through the phase transition have a higher affinity for water than the bulk water-water-water interactions, and thus these are ordered bound waters.

Protein binding sites in almost all cases do not have any bound waters when driven through the phase transition. This can be understood from the perspective that Nature needs to have ready access to binding sites to perform functional operations and that bound waters cannot be in the way, because the operations have to be reversible and repeatedly carried out. Cycles of binding and removing high affinity water molecules would be too costly energetically to be part of this function. What is very interesting, in some of our simulations on proteins that do use water in catalysis, we generally find the water bound at the functional site in the region of the inflection point of the phase transition. Thus, this water has high enough affinity to participate in functional operations, but binds with only modest affinity and thus can be readily removed.

High affinity binding of diverse fragment clusters with water exclusion is a hypothesis about the general nature of a protein interaction site. Physically, this hypothesis states that a large diversity at a localized site must exist to have enough interactions to generate specificity, and this set of interactions must readily out-compete water. Given all the ligand-macromolecule and macromolecule-macromolecule interactions that occur in nature, a combinatorial diversity of many different interactions must occur in order to attain specificity. Since water is omnipresent, we hypothesize that the interaction sites must be configured, in general, to not bind water with high affinity, because displacing high affinity waters would be too costly.

We always first use SACP to determine the sites on a macromolecule that have high affinity for water—again this is quantitatively and automatically determined by generating a dehydrating phase transition. We do the same for every fragment to find high affinity protein binding sites. By locating sites on the protein that have high affinity for a fragment cluster and only considering the sites that also do not bind water, we are explicitly determining a cluster of interactions that can easily out-compete water binding.

For all cases in this study, the fragment cluster analysis was done only at sites where water was predicted to not bind except for HIV protease, PDF, and BPTI. The reason for this is because these are water molecules that continue to bind to the protein after the phase transition and thus are the exception to the water exclusion principle. The HIV case was explicitly chosen to see if SACP correctly predicted this well-known water molecule at the center of the binding site. This was an important test, because we have run SACP on over 100 proteins and in all cases the binding sites do not retain water after the phase transition. So the fact that SACP creates a ring of waters around the outside of the HIV protease binding site in accord with the water exclusion principle, but also predicts the singular high affinity water at the center of the binding site is extremely important. HIV protease inhibitor design often incorporates protein interactions mediated by this water, so it is important that a truly predictive technique finds this water since it provides key bridging interactions with the inhibitor. We include the BPTI simulations to illustrate the need for multi-body sampling that overcomes high energy barriers and also to illustrate the nature of how water binds as a collective. Finally, the only fragment simulations that included bound waters were for PDF. In all other cases the bound waters were far away from the binding site, so they were immaterial. For PDF, however, the triplet of waters that completed the outer edge of the binding site remained after the phase transition. These waters mediate the predicted ligand binding that was experimentally confirmed.

## Conclusions

The collection of systems chosen for this study were selected to test a computational fragment-based method in a broad diversity of molecular recognition events that include electron transfer (DHFR), multiple ligand binding co-crystals that dramatically expand our knowledge of the protein binding site over time (Elastase), water-mediated recognition (HIV protease), protein-protein interactions with subtle differences in molecular recognition at the same site (p53-MDM2 versus p53-MDMX), and protein-RNA interactions (TAT-TAR of HIV). The surprising and unexpected results on two additional systems, RecA and PDF, were so intriguing that we decided to conduct fragment binding experiments to test these predictions. In all cases using SACP to uncover the high affinity locations on a macromolecule for an organic fragment or water by driving the chemical potential through a phase transition coupled with clustering and water exclusion identified the key locations responsible for molecular recognition. Taken together, these studies provide strong evidence that fragment-based techniques coupled with our three-part hypothesis is a powerful general way of investigating molecular recognition phenomena.

## Supporting information

S1 File Supporting information. This file contains the details of the biological assays, MS and NMR characterization of the small molecules described in this study. The files also contains additional information about the details of the simulations.
(PDF)

S2 File PDB data files. PDB data files containing computed fragments.
(ZIP)

S1 Fig An illustration of the thermodynamics of ligand-protein binding. The protein binding site must be dehydrated with a DG(P-H2O) as shown in the top line in order to bind a ligand. The ligand must be dehydrated with a DG(L-H2O) as shown in the bottom line in order to interact with the protein. These two lines converge in the middle with the ligand coming together with the protein DG(P-L). Fragment binding and protein hydration-dehydration are rigorously computed with Simulated Annealing of Chemical Potential (SACP). Ligand dehydration was neglected for the reasons described in the text.
(TIF)

S2 Fig Basis fragment set for hot-spot simulations. The basis fragment set used to probe the various targets described in the manuscript. This is similar to the dataset in our previous publication.
(TIF)
