# Supplementary material for: Hot-spot identification on a broad class of proteins and RNA suggest unifying principles of molecular recognition
Source: PLoS One. 2017 Aug 24;12(8):e0183327. doi: 10.1371/journal.pone.0183327 (PMC5570288; doi:10.1371/journal.pone.0183327)
Supplement: S1 File — This file contains the details of the biological assays, MS and NMR characterization of the small molecules described in this study. The files also contains additional information about the details of the simulations. (PDF) [file pone.0183327.s001.pdf]

Supporting Information

Peptide deformylase assays

Materials

Testing compounds were diluted with 10% DMSO. Actinonin was purchased from Sigma-Aldrich and will be diluted with 10% DMSO. f-Met-Ala-Ser(fMAS) was purchased from GenScript and diluted with deionized water. Fluorescamine was purchased from Sigma and diluted with dry dioxane. DMSO was purchased from Amresco.

Buffers

Buffer A, 20 mM Tris, pH 8.0, 10 mM NaCl, 5 mM $NiCl_2$; Buffer B, 20 mM Tris, pH 8.0, 5 mM $NiCl_2$.

Methods

1. The amplification of plasmid pET-22b-def and restriction enzyme analysis

The plasmid pET-22b-def obtained from Professor Pei was transformed into DH5αcompetent cells, which were incubated overnight on LB agar plates supplemented with 100 μg/ml ampicillin at 37℃. The overnight culture of DH5αcells harboring plasmid pET-22b-def was grown at 37℃ for 16h with shaking in LB medium containing 100 μg/ml ampicillin for plasmid amplication. The plasmids were extracted and confirmed by the assay of the enzyme cleavage using EcoR Ⅰ and Nde Ⅰ. It is consistent[1] with a previous report (～

500bp).

2. Expression of PDF and determination

The plasmid pET-22b-def was transformed into E.coli BL21(DE3). Clones were selected at 37℃ on LB agar plates supplemented with 100 μg/ml ampicillin. The overnight culture of BL21(DE3) cells was grown at 37℃ with shaking in LB medium containing 100 μg/ml ampicillin. The culture was transferred to the 1L LB medium containing 100 μg/ml ampicillin at 1% inoculation(The culture left was added to glycerol and stored at −80℃), and incubation was continued for 2-3h at 37℃ with shaking to get cells concentration of an OD_{600} of 0.6. The culture was added by IPTG to a final concentration of 200 μM and was induced for 4 h at 30℃. The cells were harvested by centrifugation and resuspended in buffer A plus protease inhibitor cocktail. The cells were disrupted by sonication. Cell debris was removed by centrifugation. The overexpressed PDF in BL21(DE3) cells was determined by 15% SDS-PAGE. The result is consistent[2] with a previous report. In above steps, E.coli BL21(DE3) cells and E.coli BL21(DE3) cells carrying pET-22b were used as control.

3 Purification[3] of Ni-PDF

All steps were carried out at 0-4 ℃ unless otherwise stated. The supernatant obtained was loaded onto a Q-Sepharose Fast Flow column and was eluted

with buffer A plus a linear gradient of KCl from 0 to 0.5mol/L. Fractions were collected and analysed by SDS-PAGE. The fractions containing the majority of the 20 kDa PDF were pooled. The pooled fractions were concentrated using Centrifugal Filter Devices, then was loaded onto a Sephacryl S-300(size exclusion chromatography) column. Proteins were eluted with a linear gradient of 0 to 0.15mol/L NaCl in buffer B. Fractions were collected and analysed by SDS-PAGE. The purest fractions were pooled, concentrated in Centrifugal Filter Devices and stored at -80° C. The enzyme concentration was determined using Protein Assay Kit with Bovine Serum Albumin as standard.

4 PDF in vitro Assay[4]

The assay was performed in a single 96 well plate in a final volume of 100 μl containing:

- 20 μl 0.4 μg/ml PDF
- 20 μl 400 mM Hepes pH 7.0+3.5M KCl+0.175% Brij+5mM $NiC1_2$
- 10 μl serial dilution of test compound in 10% DMSO
- 50 μl 8mM formyl-Met-Ala-Ser

The assay was incubated at 37° C for 0, 0.5, 1, 2, 4, 8, 24 h, respectively. The free amino group of the deformylated (Met-Ala-Ser) product was detected using fluorescamine, by the following additions:

- 50 μl 0.2M borate pH 9.5
- 50 μl fluorescamine (200 μg/ml in dry dioxane)

Fluorescence was quantified on Thermo Fluoroskan Ascent FL using an excitation wavelength of 390 nM and an emission wavelength of 485 nM. Standard control reactions are a no inhibitor reaction which provides the zero inhibition figure and a no enzyme and no inhibitor reaction which provides the 100% inhibition figure. The data was analysed by conversion of the fluorescence units to % inhibition and the inhibitor concentration plotted against % inhibition. The data was fitted to a sigmoidal function: $y=A+((B-A)/(1+((C/x)^D)))$, wherein A represents zero inhibition, B represents 100% inhibition and C represents the IC_{50}, D represents the slope. The IC_{50} represents the concentration of inhibitor required to decrease enzyme activity by 50%.

NMR and MS Characterization for PDF Inhibitors

Indole derivatives were synthesized as previously described.[5]

N-hydroxy-2-(1H-indol-2-yl)acetamide (2)

^{1}H NMR (300 MHz, CH_3OD) δ 10.91 (br s, 1H), 10.62 (br s, 1H), 8.87 (br s, 1H), 7.38 (d, 1H, J = 6.0 Hz), 7.28 (d, 1H, J = 6.0 Hz), 6.97 (t, 1H, J = 6.0 Hz), 6.89 (t, 1H, J = 6.0Hz), 6.16 (s, 1H), 3.42(s, 2H). LCMS 191.3 $[M\ ]^+$.

N-hydroxy-2-(1-methyl-1H-indol-3-yl)acetamide (3)

^{1}H NMR (300 MHz, CH_3OD) δ 10.57 (br s, 1H), 8.72 (br s, 1H), 7.54 (d, 1H, J =

6.0 Hz), 7.34 (d, 1H, J = 6.0 Hz), 7.13 (s, 1H), 7.12 (t, 1H, J = 6.0 Hz), 6.98 (t, 1H, J = 6.0 Hz), 3.33 (m, 3H) 2.47 (s, 2H). LCMS 204.1 $[M]^+$.

N-hydroxy-2-(1-isopropyl-1H-indol-3-yl)acetamide (4)

^{1}H NMR (300 MHz, CH_3OD) δ 10.57 (s, 1H), 8.74 (br s, 1H), 7.53 (d, 1H, J = 6.0 Hz), 7.41 (d, 1H, J = 6.0 Hz), 7.27 (s, 1H), 7.07 (t, 1H, J = 5.7 Hz), 6.98 (t, 1H, J = 5.7 Hz), 4.65 (dt, J = 5.1 Hz), 2.47 (s, 2H), 1.39 (d, 6H, J = 5.1 Hz). LCMS 232.2 $[M]^+$.

Simulation Details

All simulations place the protein in a rectangular box large enough to accommodate three layers of solvent and are done with periodic boundary conditions. These two operations minimize artifacts that can arise from edge effects.

The grand canonical Monte Carlo (GCMC) algorithm used in this study was developed by Adams[5] in 1975. In the previous studies done by Guarnieri and co-workers, Mezei's cavity-bias[6] technique with detailed balance corrections to improve the efficiency of the Adam's algorithm was used. In this study, however, we do not use cavity-bias, instead it has been replaced with a simple biased learning algorithm that is much easier to implement and maintain during

the simulation. To our knowledge, since there is no description in the literature of the technical difficulties in performing cavity-biased GCMC, we will describe these difficulties here, which will clearly show the motivation for developing a new method.

The cavity-bias method requires creating a 3-dimensional grid inside the protein simulation cell. An algorithm systematically scans every grid point to discover the vertices that are occupied by a protein atom and the vertices that are not. Fragment insertion is attempted only where there are free grid points. The cavities are continuously monitored during the simulation, because when a fragment insertion is accepted, the previously free grid is now occupied and no further insertion attempts occur at this site unless a successful deletion from this site happens. There are 2 major practical problems with cavity-bias, 1) the appropriate spacing of the grids is not obvious and 2) where the grids are started is arbitrary. If the grid spacing is made very small, <1A for example, then there is a very large number of grids to keep track of during the simulation and many of them are superfluous. If the grid spacing is made larger, there is a chance of missing an important cavity. When it was recognized that important cavities could be missed in this process, an algorithm that periodically shifts the grids by a fraction of an Angstrom was employed, because repositioning the grids often uncovers an additional cavity. These procedures result in several difficulties, 1) how often should the grids be

shifted, 2) once the grids are shifted how many new and additional trial configurations need to be generated to sample potentially newly uncovered cavities, 3) many of the grids are superfluous arising from an overly dense grid due to the desire to not miss anything important, 4) an overly dense grid significantly slows down the simulation making it much less efficient, and 5) what is the best way to keep track of the difference between multiple sets of shifted grids. To avoid all of these issues, the grid spacing is made very dense by setting it to half of an Angstrom, which requires no grid shifting. Because every fragment is significantly larger than half of an Angstrom, an algorithm is needed to determine where a set of connected free grid points of sufficient volume is located in order to find viable cavities for attempted particle insertion. This is done for every fragment, since different fragments have different sizes.

All of these considerations require maintaining, and updating a large bookkeeping operation during a GCMC simulation. Since GCMC is run at one fixed chemical potential while SACP is a sequence of chemical potentials, SACP with cavity bias requires following the grids through a whole multiplicity of such simulations.

Intrinsic to the SACP process is a clear method for replacing cavity-bias with a simple learning algorithm. GCMC simulations at high chemical potentials overcome energy barriers and there is a dramatically enhanced probability of

accepting an inserted trial fragment at these high chemical potentials except if the attempted insertion directly overlaps with a protein atom. Fragment overlap with a protein atom can be readily and quickly detected by doing a vectorized Lennard-Jones calculation with SIMD vector instructions on 4-vectors available in the Intel architecture. This allows for detecting 4 different potential atom overlaps simultaneously. When any atom of a trial inserted fragment overlaps a protein atom an overflow results in an IEEE NAN (not a number). This fragment is immediately rejected and a new trial insertion or deletion is attempted. Within 500,000 trial steps at very high chemical potentials the simulation cell is completely stuffed with fragments in all three solvent layers and in every possible cavity of the protein. We continue to run the simulation at this highest chemical potential until the particle number changes by <1% for 200,000 steps. Just to be clear, the particles are randomly inserted and deleted with equal probability anywhere within the simulation cell. The fast rejection when atom overlap occurs and the initial very high chemical potential results in fragments being inserted in every protein cavity capable of accommodating a fragment and with a completely filled bulk.

When the chemical potential is lowered (this is the annealing phase) new trial inserts and deletes actually produce little to no change in the system. This is because the large multi-body network of interactions between the densely

packed solvent particles is extremely stable and resistant to a fragment deletion, which would result in small evacuated areas. Insertions are not possible, because every part of the simulation cell is packed with fragments. There is only a significant change in the system when the chemical potential is lowered to a free energy value that is capable of destroying the large connected network of solvent-solvent-solvent multibody interactions. For different fragments the position of the phase transition will be different so we initially run an SACP simulation lowering the chemical potential in a very coarse manner to approximately locate the phase transition region. The coarse chemical potential schedule that we typically use is: 100, 50, 20, 10, 5, 2, 0, -2, -5, -10, -20, -50, and -100. Simulations can be run by setting a fixed number of trial configurations, such as 5,000,000. To avoid an arbitrary choice, we simply monitor the average number of particles at a given chemical potential and when this particle number changes by <1% for 200,000 trials, the simulation is deemed to have reached equilibrium and it goes to the next lower chemical potential with the process repeated. When the particle number changes by greater than 40% between 2 different chemical potentials, the phase transition has been located and this preliminary phase is automatically ended.

The real simulation starts with a check point file at a high enough chemical potential that is just before the phase transition. The annealing schedule is

set to decrease the chemical potential by a single digit. For example, if the phase transition occurs between -5 and -10, the simulation is restarted with a chemical potential of -5 using the final configuration from the check point file and the annealing schedule is -6, -7,-8,-9 …etc. Just to be clear, since -5 is before the phase transition the simulation box is completely stuffed with fragments including every possible internal protein cavity capable of binding a fragment. As the simulation proceeds with lowering the chemical potential, deletion attempts becomes more probable and occur more often. Every time a fragment is deleted that is anywhere in the vicinity of the protein including any internal cavities, its coordinates are recorded in a table. This provides a detailed and easy record of all the cavities in the protein capable of binding a fragment with no superfluous cavities, no need for grids, and thus no need for grid shifting. When the simulation reaches a chemical potential where the number of total particles in the simulation cell has dropped significantly, this indicates that much of the bulk particles have been deleted. This means that trial insertions into the bulk will almost completely fail, because it is attempting to insert a particle into a vacuum at a relatively low chemical potential. This is when surface insertion bias is turned on.

Specifically, as the annealing process continues, surface insertion bias is turned-on when one of two criteria is met whose purpose is to detect that the simulation is in a surface-only mode (no fragments in the bulk):

1. The average over all fragments of the minimum distance between the center of mass of the fragment and the center of mass of the nearest protein residue is less than the sum of the fragment radius and the average of all the protein residue radii. The radius in this case is defined as the distance of the atom furthest from the center of mass, plus its van der Waals radius. And the number of fragments remaining in the system is less than the number of protein residues (so the criterion scales with protein size). Note that this criterion takes advantage of the fact that fragments and residues are not spherical and have an aspect ratio more than 1 so that fragments must be tightly bound to the protein to achieve the criterion. This is questionable for small fragments like ammonia, methane, or water. In those cases the second criterion kicks in.
2. The number of fragments remaining in the system is less than a fixed number (typically, 100). This criterion works fine and has the advantage of being very simple.

When surface insertion bias is active, each time a fragment is deleted its coordinates are added to a list. This algorithm is extremely simple and exceptionally accurate – a location in the protein is retained on a list of cavities only if a fragment has been successfully deleted from this position, thus there are no spurious cavities sampled during this phase of the simulation. This list

serves as location bias sites for subsequent insertions. At the beginning of each new lower chemical potential, sites that have less than 50% occupancy during the previous higher chemical potential sampling period are removed from further consideration. A biased sampling technique requires detailed balance corrections to maintain microscopic reversibility. At any particular point in the simulation some of the cavities in the list are occupied by a fragment and some are free. As mentioned above, when we are in the first phase of the annealing schedule with random insertions and deletions, thus requiring no detailed balance corrections, the GCMC probability density function as formulated by Adams is used for the accept-reject criteria. In the surface insertion bias phase the detailed balance correction for attempted insertions is NFREE/(NOCCUPIED+1) and for deletions is NOCCUPIED/(NFREE-1). Two additional points should be noted, 1) when an insertion is attempted, the fragment is randomly rotated and translated by some small amount before the attempted insertion, 2) a quarter of the trial steps are canonical Monte Carlo – fragments in the simulation cell are chosen at random and randomly translated and rotated by small amounts, which is accepted or rejected with a Boltzmann probability density function.

Our compute facility is a 60-core cluster with Intel E5640 Xeon (Nehalem) 2.67GHz processors. Each fragment simulation normally runs on a single core, although there is an option to run a single simulation with up to 12 cores

using OpenMP. The simulations on elastase (1ELA) ran from 45 hours for water runs (the slowest) to 15 hours for propane (fastest). For a typical clustering set of 20 fragments and waters, approximately 450 core-hours were required. In 2017, on Amazon Web Services with C4 (Skylake) instances, plus further optimization of the simulation code, this has come down to about 125 core-hours (at a cost of roughly $0.01/core/hour for spot instance pricing). The scaling is highly dependent on protein size. When a binding site is known, a protein subset can be run with location biased sampling to minimize run time to numbers similar to elastase. When searching for all hot-spot binding spots on a large protein complex, the run time can be 5-10 times larger. On AWS we typically can access 1,000 cpu's with 36 cores per cpu, so the hot-spot computations are inexpensive ($10-$20) and can be generally done in 1-3 days.

Details of the Force Fields

The force field parameters used for the protein[7] were Amber94. The fragment van der Waals parameters were from Gaff[8] data. The partial charges for the fragments were generated using Firefly[9] 8.01 using a 6-31G* basis with DFT B3LYP to generate quantum mechanical electron distributions that were then used to generate partial charges with RESP[10] fitting. The water parameters used in this study were developed in the Jorgensen[11] lab.

TIP3 parameters were used for the water-protein interactions and TIP4 parameters were used for the water–water interactions.

Details of the Thermodynamic Cycle

The process of fragment binding to a protein can be broken down into the, 1) free energy of desolvating the part of the fragment that interacts with the protein, 2) the free energy of desolvating the sub-pocket of the protein binding site that interacts with the part of the fragment that actually binds, and 3) the free energy of this fragment-protein interaction (illustrated in S1 Fig). The present study did not need to include fragment desolvation, which can be explained by briefly summarizing one of our recent studies that did include the fragment solvation energy.

Figure S1. An illustration of the thermodynamics of ligand-protein binding. The protein binding site must be dehydrated with a DG(P-H2O) as shown in the top line in order to bind a ligand. The ligand must be dehydrated with a DG(L-H2O) as shown in the bottom line in order to interact with the protein. These two lines converge in the middle with the ligand coming together with the protein DG(P-L). Fragment binding and protein hydration-dehydration are rigorously computed with Simulated Annealing of Chemical Potential (SACP). Ligand dehydration was neglected for the reasons described in the text.

The SAMPL3 challenge of 2012 was a contest designed to evaluate the predictive abilities of fragment-based methods. The scientists running the contest picked a protein and performed X-ray analysis with fragment soaking experiments to explicitly determine all of the positions on the protein where fragment binding occurs and also conducted binding experiments to obtain the interaction free energy between the fragments and the protein. This information was kept confidential. The PDB file of the protein and the list of fragments were made available to the research community. The challenge was to submit detailed predictions of fragment binding locations and binding free energies by a deadline and the experimental data were subsequently released so that the results could be objectively judged. SACP was used to predict fragment-protein binding and water-protein binding. Clustering high affinity fragments with water exclusion readily identified the binding site. The rank order binding free energy based on the chemical potential – fragments that still remain bound as the chemical potential was lowered are predicted to have higher affinity than fragments vacating the binding site at that same chemical potential – very accurately tracked a large majority of the experimental data.

The predictions were made even more accurate when we included a continuum solvent model for the ligand dehydration. As would be expected, this was especially true when comparing fragments that are chemically and

physically very different, because the solvation free energies are very different. The continuum solvent model used in this SAMPL3 study was developed[12] in our group. While there are many models that accurately predict small molecule free energies of solvation, many of which are cited in this paper from our group, we created a new model that is of high accuracy, but is also fast enough to be incorporated into the simulations. Just to be clear, running the full annealing schedules can require performing over 100,000,000 Monte Carlo trial steps, so incorporating a continuum model for ligand solvation-desolvation free energy must be very fast. Complete details of applying SACP to the SAMPL3 challenge[13] have been published.

It was not necessary to use the continuum model in the studies presented in this paper, because the 3 molecules designed to inhibit peptide deformylase are all congeners of the same core structure and thus would have virtually identical solvation free energies. Incorporating the continuum calculation would not have altered the rank-order binding predictions, so there was no need to introduce this added calculation. For the RecA studies, the SACP simulations led to the clear prediction of one molecule, 6-hydroxydopamine, which we purchased and had tested. The 6-HD will obviously be completely and strongly water solvated, because of the positively charged amine group and the 3 hydroxyl groups Additionally, because no comparative analysis between different fragments was required, there was no need to employ the

solvation free energy calculation for 6-HD. The SACP prediction was so compelling because the fragment pattern produced 6-HD interacting with RecA in a manner that mimicked 2 universally conserved amino acids, so it was worth just testing it experimentally. Finally, for all of the other test cases, SACP predictions of fragment-protein and water-protein binding with clustering and water exclusion (which accounts for fragment-water competition for binding to a location on the protein) reproduced all of the known experimental data cited in this study.

Fragment set for clustering and methodology

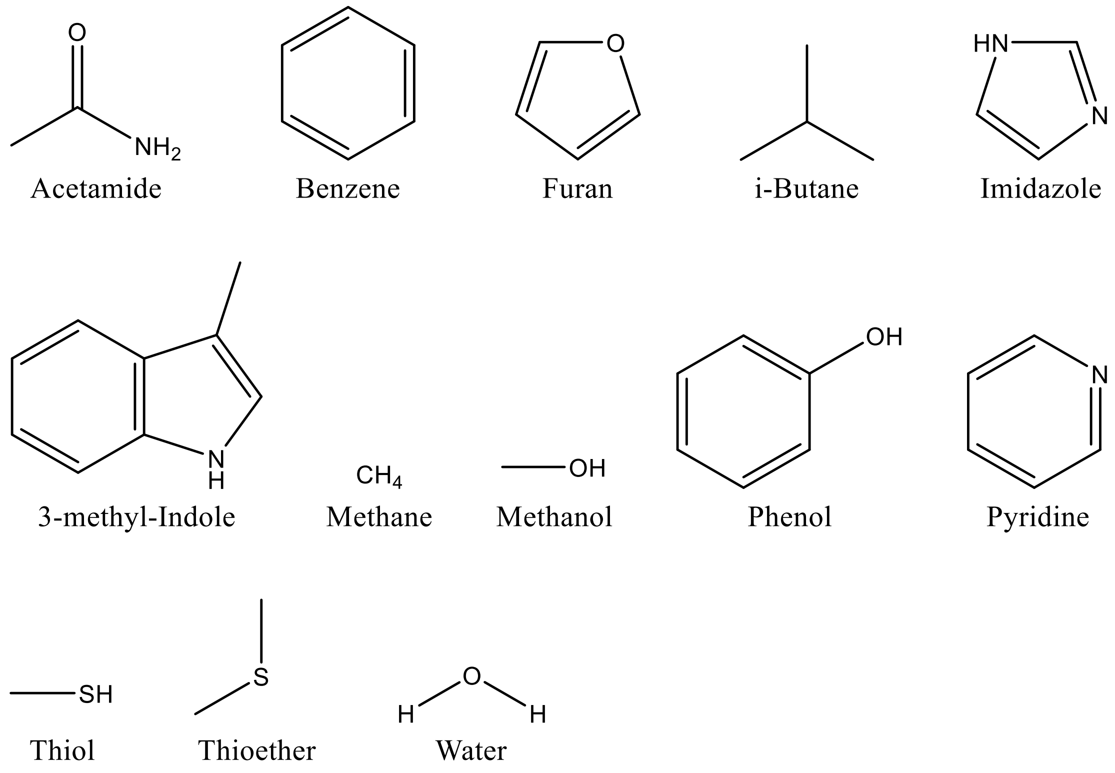

Figure S2. The basis fragment set used to probe the various targets described in the manuscript. This is similar to the dataset in our previous publication.[14]

Successful computational hot-spot mapping using are algorithm is based on three principles. First, the chemical fragments need to have highly favorable interaction energies with the protein. If the energies are too favorable, there will be one or no fragment binding sites. On the other hand, if the energies are marginally favorable, say at the phase transaction, then the entire protein surface will be covered with fragments. Thus, we take energetic values approximately halfway between the phase transition and the value at which

only one fragment appears on the surface. Our second principle is to exclude sites where tightly bound water molecules could potentially block fragment binding. Our SACP approach gives quantitative values for each water molecule on the protein surface. We categorize these into three bins (i) bulk water like and easy to displace, (ii) bound but only a small energy to displace, and (iii) tightly bound and high energetic cost to replace. During our clustering algorithm, the waters in category iii are kept in place and exclude hot-spot identification in those sites. Lastly, we look for sites where a diverse set of chemical fragments bind.

References

1. Rajagopalan PT, Datta A, Pei D. Purification, characterization, and inhibition of peptide deformylase from Escherichia coli. Biochemistry. 1997;36(45):13910-8. PubMed PMID: 9374870.
2. Rajagopalan PT, Pei D. Oxygen-mediated inactivation of peptide deformylase. J Biol Chem. 1998;273(35):22305-10. PubMed PMID: 9712848.
3. Xian-bing T, Shu -y S, Yue-qin Z. Establishment of a high throughput screening model targeted on peptide deformylase (PDF) of E. coli. Chinese Journal of Antibiotics. 2003;28:621-6.
4. Clements JM, Beckett RP, Brown A, Catlin G, Lobell M, Palan S, et al. Antibiotic activity and characterization of BB-3497, a novel peptide deformylase inhibitor. Antimicrob Agents Chemother. 2001;45(2):563-70. PubMed PMID: 11158755.
5. Adams DJ. Grand canonical ensemble Monte Carlo for a Lennard-Jones fluid. Mol Phys 1975;29(1):307-11.
6. Mezei M. Grand-canonical ensemble Monte Carlo study of dense liquid Lennard-Jones, soft spheres and water. Mol Phys. 1987;61:565-82.
7. Cornell WD, Cieplak P, Bayly CI, Gould IR, Merz KM, Ferguson DM, et al. A Second Generation Force Field for the Simulation of Proteins, Nucleic Acids, and Organic Molecules. Journal of the American Chemical Society. 1995;117(19):5179-97.
8. Wang J, Wolf RM, Caldwell JW, Kollman PA, Case DA. Development and testing of a general amber force field. J Comput Chem. 2004;25(9):1157-74. PubMed PMID: 15116359.
9. Schmidt MW, Baldridge KK, Boatz JA, Elbert ST, Gordon MS, Jensen JH, et al. General Atomic and Molecular Electronic Structure System J Comput Chem. 1993;14(11):1347-63.
10. Steinbrecher T, Latzer J, Case DA. Revised AMBER parameters for bioorganic phosphates. J Chem Theory Comput. 2012;8(11):4405-12. PubMed PMID: 23264757.
11. Jorgensen WL, Chandrasekhar J, Madura JD, Impey RW, Klein ML. Comparison of simple potential functions for simulating liquid water. The Journal of Chemical Physics. 1983;79(2):926-35.
12. Boyer RD, Bryan RL. Fast estimation of solvation free energies for diverse chemical species. J Phys Chem B. 2012;116(12):3772-9. PubMed PMID: 22339050.
13. Kulp JL, 3rd, Blumenthal SN, Wang Q, Bryan RL, Guarnieri F. A fragment-based approach to the SAMPL3 Challenge. J Comput Aided Mol Des. 2012;26(5):583-94. PubMed PMID: 22290624.
14. Kulp JL, 3rd, Kulp JL, Jr., Pompliano DL, Guarnieri F. Diverse fragment clustering and water exclusion identify protein hot spots. J Am Chem Soc. 2011;133(28):10740-3. PubMed PMID: 21682273.
