# Supplementary figures and images for: Hot-spot identification on a broad class of proteins and RNA suggest unifying principles of molecular recognition
Source: PLoS One. 2017 Aug 24;12(8):e0183327. doi: 10.1371/journal.pone.0183327 (PMC5570288; doi:10.1371/journal.pone.0183327)

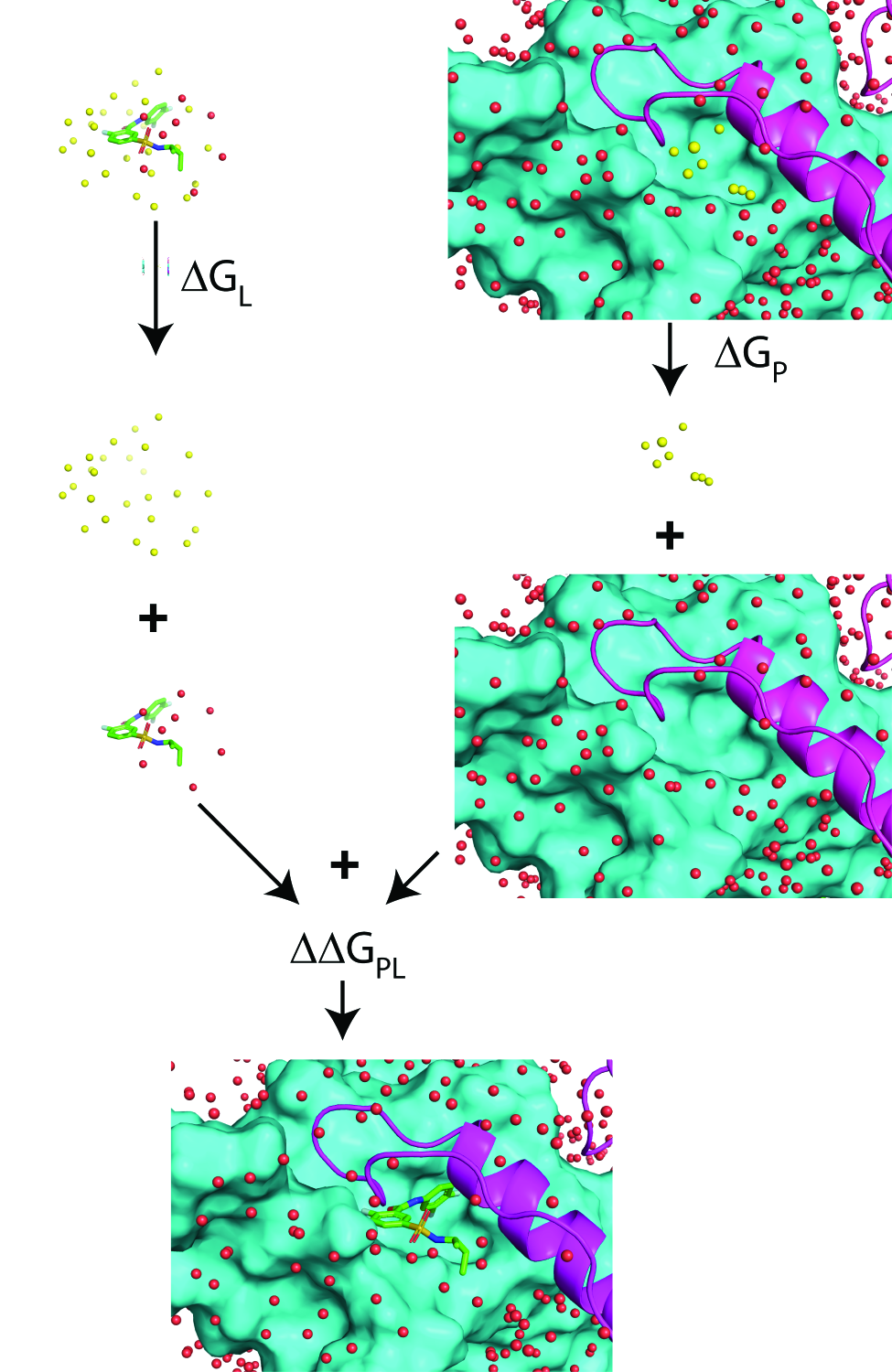

Supplement: S1 Fig — The protein binding site must be dehydrated with a DG(P-H2O) as shown in the top line in order to bind a ligand. The ligand must be dehydrated with a DG(L-H2O) as shown in the bottom line in order to interact with the protein. These two lines converge in the middle with the ligand coming together with the protein DG(P-L). Fragment binding and protein hydration-dehydration are rigorously computed with Simulated Annealing of Chemical Potential (SACP). Ligand dehydration was neglected for the reasons described in the text. (TIF) [file pone.0183327.s003.tif]
